# Stat3/IL-6 signaling mediates sustained pneumonia induced by *Agiostrongylus cantonensis*

**DOI:** 10.1371/journal.pntd.0010461

**Published:** 2022-05-26

**Authors:** Hongli Zhou, Yuting Lu, Hang Wei, Yixin Chen, Yanin Limpanon, Paron Dekumyoy, Ping Huang, Peiyao Shi, Zhiyue Lv

**Affiliations:** 1 Key Laboratory of Tropical Disease Control (Sun Yat-Sen University), Ministry of Education, Guangzhou, China; 2 Key Laboratory of Tropical Translational Medicine of Ministry of Education, Hainan Medical University, Haikou, China; 3 Faculty of Tropical Medicine, Mahidol University, Bangkok, Thailand; 4 Department of Experimental Diagnosis, Shenzhen Yantian District People’s Hospital, Guangdong, China; 5 Department of Infectious Disease, Hainan Affaliated Hospital, Hainan Medical University, Haikou, China; University of Passo Fundo: Universidade de Passo Fundo, BRAZIL

## Abstract

*Angiostrongylus cantonensis* (AC) is well-documented that parasitizes the host brain and causes eosinophilic meningitis. The migration route of AC in permissive hosts is well demonstrated, while in nonpermissive hosts, it remains to be fully defined. In the present study, we exploited live imaging technology, morphological and pathological configuration analysis, and molecular biological technologies to explore the migration route of AC and the accompanying tissue damage in nonpermissive and permissive hosts. Our data indicated that, in nonpermissive host mouse, AC larvae migrated from intestinal wall to liver at 2 hours post-infection (hpi), from liver to lung at 4 hpi and then from lung to brain at 8 hpi. AC larval migration caused fatal lung injury (pneumonia) during acute and early infection phases, along with significant activation of Stat3/IL-6 signaling. In addition, AC induce sustained interstitial pneumonia in mouse and rat and pulmonary fibrosis only in rat during late infection phase. Moreover, during the early and late infection phases, Th2 cytokine expression and Stat3 and IL-6 signaling were persistently enhanced and myeloid macrophage cells were notably enriched in host lung, and administration of Stat3 and IL-6 inhibitors (C188-9 and LMT-28) attenuated AC infection-induced acute pneumonia in mice. Overall, we are the first to provide direct and systemic laboratory evidence of AC migration route in a nonpermissive host and report that infection with a high dose of AC larvae could result in acute and fatal pneumonia through Stat3/IL-6 signaling in mice. These findings may present a feasible to rational strategy to minimize the pathogenesis induced by AC.

## Author summary

*Angiostrongylus cantonensis* is a well-known nematode parasitizing the host brain and is the leading cause of eosinophilic meningitis worldwide. Our study reports the migration route of *Angiostrongylus cantonensis* larvae in non-permissive host mouse and discovers that the larvae could induce fatal pneumonia in mouse lung during acute and early infection phase characterized by activation of Stat3/IL-6 signaling. Moreover, administration of inhibitors targeting Stat3/IL-6 signaling could significantly attenuate the AC-induced pneumonia of non-permissive host mouse, providing an effective candidate target for intervention of this severe parasitic pneumonia. These findings expand our understanding of *Angiostrongyliasis cantonensis* and indicate that more attention to AC-induced pneumonia is required when treatment for *Angiostrongyliasis cantonensis* in the future.

## Introduction

Parasitosis is attributed to infection by parasites, which can cause host disease directly by migration from one organ to another in the body [1,2] or indirectly by the toxins they produce [3]. *Angiostrongylus cantonensis* (AC) is a food-borne nematode that infects hosts via ingestion of foods containing infective third-stage larvae of AC (AC L3) [4]. Based on the different pathological outcomes, AC hosts are separated into two types: permissive hosts (such as rats) and nonpermissive hosts (such as mice and humans) [5]. In permissive host rats, AC migrates from the intestinal wall to the liver within 2 hours post-infection (hpi), to the lung at 2 hpi and the brain at 8 hpi [6] and finally returns from the brain to the lung at 28 days post-infection (dpi) [7] where adult worms lay eggs [8]. During the process of migration in rats, it is well documented that AC causes optic neuritis [9], eosinophilic meningitis [10] at 21 dpi and calcified pulmonary nodules during the late phase of infection [11]. However, whether AC causes damage to other tissues in rats during the early infection phase (from 1 to 7 dpi) and late infection phase (from 7 to 42 dpi) still needs more exploration. In contrast, in nonpermissive host mice, AC could lead to fatal eosinophilic meningitis during the late infection phase (at 21 dpi) [12]. In another nonpermissive host, humans, a rare case of AC adult worms present in the lung tissue was reported in a 5-year-old female patient who died from angiostrongyliasis cantonensis [13], indicating that the lung tissue was also damaged. However, to date, researchers have mainly focused on eosinophilic meningitis but neglected lung damage caused by AC infection. Regarding the characteristics of pulmonary pathological injuries, the underlying mechanism, the specific route and corresponding timepoint of AC migration in nonpermissive hosts throughout the infection phase remain to be fully elucidated. This work will provide new insights into angiostrongyliasis cantonensis and provide new opportunities for therapy.

Conventional pneumonia is characterized by fluid or pus filling the air sacs and is commonly attributed to pathogen infections, including infections caused by bacteria, viruses, fungi and parasites [14]. Interstitial pneumonia is another nonspecific pneumonia without fluid present in the air sacs but with inflammation or fibrosis in the alveolar wall [15]. Parasitic pneumonia can be induced by a number of parasites, including *Ascaris*, hookworms, *Strongyloides*, *Paragonimus filariasis*, and *Toxocara* [16]. Some nematode parasites, such as *Strongyloides*, penetrate the host lung, while others, such as *Trichuris*, do not. Generally, parasites can cause pneumonia through direct stimulation or cytokine-induced systemic inflammation [17]. It has been reported that the parasite *Ascaris* could cause a severe eosinophilic reaction, which may lead to eosinophilic pneumonia [16]. As AC is known to induce meningitis characterized by eosinophilic reactions and AC might migrate through the host lung, we asked whether AC infection could result in conventional or interstitial pneumonia in the host. If so, we aimed to elucidate the characteristics of and potential intervention targets for AC-induced pneumonia.

Many studies have reported that parasite infection triggers the host immune response, mainly the Th1 and Th2 responses [18]. The Th1 response is characterized by elevated expression of the cytokines TNF-α, IL-1β, IFN-γ and INOS [18–21], while the Th2 response is characterized by elevated expression of IL-4, IL-6, IL-10 and IL-13 [18,22,23]. In addition, Th1 and Th2 cytokines are closely associated with the progression of pneumonia [24]^,^ and hosts infected with AC often exhibit a shift from a Th1 to Th2 response [25]. Furthermore, innate immune cells (such as myeloid and dendritic cells) and adaptive immune cells (such as T cells) are involved in host resistance to pathogens. However, during the process of AC infection, especially the acute and early infection phases, how AC worms affect the immune response (immune cell activation and cytokine expression) in parasitized organs of the host remains largely unknown. Therefore, in this study, we aimed to investigate these questions and expand our understanding of parasitosis induced by AC.

In the present study, we used live imaging technology to explore the migration route of AC in nonpermissive host mice, revealing that after infection, AC L3 migrated from the intestinal wall to the liver at 2 hpi, then to the lung at 4 hpi and the brain at 8 hpi. Surprisingly, we observed fatal pneumonia in the mouse lung during the acute and early phases of AC infection. In addition, AC could lead to sustained slight interstitial pneumonia during the late infection phase. Moreover, the immune response of the host to AC exhibited a Th2 bias characterized by activation of Stat3/IL-6 signaling and enrichment of innate immune myeloid cells. Inhibitors targeting Stat3 or IL-6 could significantly attenuate the pneumonia induced by AC, expanding our understanding of angiostrongyliasis cantonensis and providing effective candidate targets for intervention in AC-induced pneumonia.

## Methods and materials

### Ethics statement

All research and animal care procedures were performed according to protocols approved by the Institutional Animal Care and Use Committee of Sun Yat-Sen University.

### Parasite preparation and labeling

Third-stage larvae of AC (AC L3) were obtained from positive *Biomphalaria glabrata* (*B*. *glabrata*), as described previously [26]. Briefly, *B*. *glabrata* was homogenized and digested for 30 minutes at 37°C with pepsin solution. After that, AC L3 were counted under a stereomicroscope (SZ650, China) and placed in a 1.5 mL centrifuge tube with 100 μL distilled water prior to animal infection. To trace the migration route of AC in mice, AC L3 were washed with saline followed by incubation with the green fluorescent dye Nuclear Green LCS1 (AAT Bioquest, United States) for 1 hour at room temperature. Then, AC L3 were washed three times with saline before fluorescence imaging. The labeling efficacy was assessed by imaging under a fluorescence microscope.

### Tracing the migration route of AC L3 in mice

The mice were infected with 100 to 300 AC L3 labeled with green fluorescence by oral gavage and sacrificed at 0-, 2-, 4-, 8- and 12-hours post-infection followed by removal of the liver, lung and brain. Live imaging was used to test the existence of AC L3 in specific tissues under a living imager (PerKinElmer, United States). After that, the liver, lung and brain were excised and homogenized to determine AC L3 counts under a stereomicroscope (SZ650, China).

### Animals

Female six-week-old C57BL/6 mice and Sprague–Dawley (SD) rats were purchased from Charles River Laboratories (Beijing, China) and randomly divided into the mentioned groups, and 5 animals each group. Each mouse and rat in the following study was infected with 30 and 100 AC L3, respectively, by oral gavage. At the experimental endpoint, mice and rats were anesthetized and sacrificed for the following experiments. All animals were housed in a specific pathogen-free, temperature-controlled environment with a 12-hour light/dark cycle. All animal procedures were approved by the Institutional Animal Care and Use Committee of Sun Yat-sen University.

### H&E and Masson staining

To examine the histomorphological configuration alterations caused by AC L3 migration in mice, the livers, lungs and brains were removed after AC infection, fixed in formalin (4% paraformaldehyde), and embedded in paraffin. Tissues were sliced into 4-μm serial sections and stained with hematoxylin and eosin (H&E) or Masson trichrome according to reported previously methods [4]. The stained sections were imaged under a microscope, and the fibrotic area was analyzed using Fiji ImageJ (NIH) software.

### Quantitative real-time PCR

Total RNA from the lung or brain was extracted with the use of Trizol Reagent (Invitrogen, Carlsbad, CA) following the manufacturer’s instructions. The concentration and purity of RNA were determined with a NanoDrop One (Thermo Fisher Scientific, Waltham, USA). Complementary DNA (cDNA) was synthesized by using a Revert Aid First Strand cDNA Kit (Thermo Fisher Scientific, Waltham, USA) according to the manufacturer’s guidelines. RT–qPCR was carried out using SYBR Green (TaKaRa, Dalian, China) on a LightCycler 480 Real-Time PCR System (Roche Diagnostics, Reinach, Switzerland). RT-qPCR was performed for 10 minutes at 95°C followed by 40 cycles at 95°C for 15 seconds and 60°C for 1 minute. Relative mRNA levels were normalized to the housekeeping gene β-actin and analyzed by the 2^-ΔΔCt^ method. The primers used in this study are listed in S1 and S2 Tables.

### Western blotting

Tissue samples were homogenized and lysed with RIPA lysis buffer (Thermo Fisher Scientific, USA) containing protease and phosphatase inhibitor cocktail (Thermo Fisher Scientific, USA) on ice for 5 minutes, followed by centrifugation at 12000 rpm for 15 minutes. The supernatant was collected and subjected to protein quantification with a bicinchoninic acid (BCA) kit (Beyotime, Wuhan, China). A total of 30 μg protein was separated by sodium dodecyl sulfate–polyacrylamide gel electrophoresis (SDS–PAGE) and transferred onto polyvinylidene fluoride (PVDF, 0.22μm) membranes (Merck Millipore, MA, USA), which were blocked with 5% nonfat skim milk for 2 hours at room temperature before overnight incubation with primary antibodies (S3 Table) at 4°C. After that, HRP-coupled secondary antibodies were applied, and signals were detected with an enhanced chemiluminescence (ECL) kit (Merck Millipore, MA, USA) under a ChemiDoc Imaging System (Bio–Rad, California, USA). β-Actin was used as an internal reference gene.

### Immunofluorescence

The lung tissues were fixed for 24 hours, embedded in paraffin and cut into 4-μm serial sections. For immunofluorescence, the sections were deparaffinized and subjected to antigen retrieval at 100°C for 15 minutes in citrate buffer (10 mM, pH 6.0). Next, the sections were blocked with 3% bovine serum albumin (BSA) for 1 hour at room temperature prior to primary antibody incubation at 4°C overnight. Subsequently, sections were incubated for 1 hour at room temperature with fluorescence-labeled secondary antibodies as listed in S3 Table, and 4’,6-diamidino-2-phenylindole (DAPI) was used to stain the cell nuclei. Thereafter, the fluorescence intensity was measured by confocal microscopy (LSM 880, Zeiss).

### Flow cytometry

To evaluate the apoptosis of lung cells caused by AC L3 migration, flow cytometry was carried out. The lung tissues were homogenized on ice and filtered with a 40-μm cell filter to prepare a single-cell suspension. The prepared cells were washed twice with cold PBS, followed by staining with APC-labeled annexin V and propidium iodide (PI) for 30 minutes at 4°C in the dark. Then, the cells were washed twice and further analyzed on a CytoFLEX flow cytometer (Beckman Coulter, Atlanta, USA).

### Immunohistochemistry (IHC)

Consecutive formalin-fixed and paraffin-embedded lung tissue sections were subjected to immunohistochemical staining as previously described. In brief, the sections were blocked with 10% fetal bovine serum for 1 hour, followed by incubation with primary antibody overnight at 4°C. After washing with PBS three times, sections were incubated with 3% H_2_O_2_ prior to incubation with an HRP-labeled secondary antibody and diaminobenzidine (DAB) for color development. Next, hematoxylin was used to counterstain the cell nucleus, and the sections were dehydrated, mounted with neutral gum and photographed with the use of an inverted microscope (Leica, Heidelberg, Germany).

### Statistical analysis

Statistical analyses were performed with GraphPad Prism 7.00 (San Diego, CA, USA). All data are displayed as the mean ± standard derivation (SD) of no less than three independent replicates. Significance was determined with a two-tailed Student’s *t test* or one-way analysis of variance (*P < 0.05, **P < 0.01, ***P < 0.001 and ****P < 0.0001).

## Results

### AC L3 migration through the mouse lung leads to pneumonia during the acute infection phase

**Fig 1:**
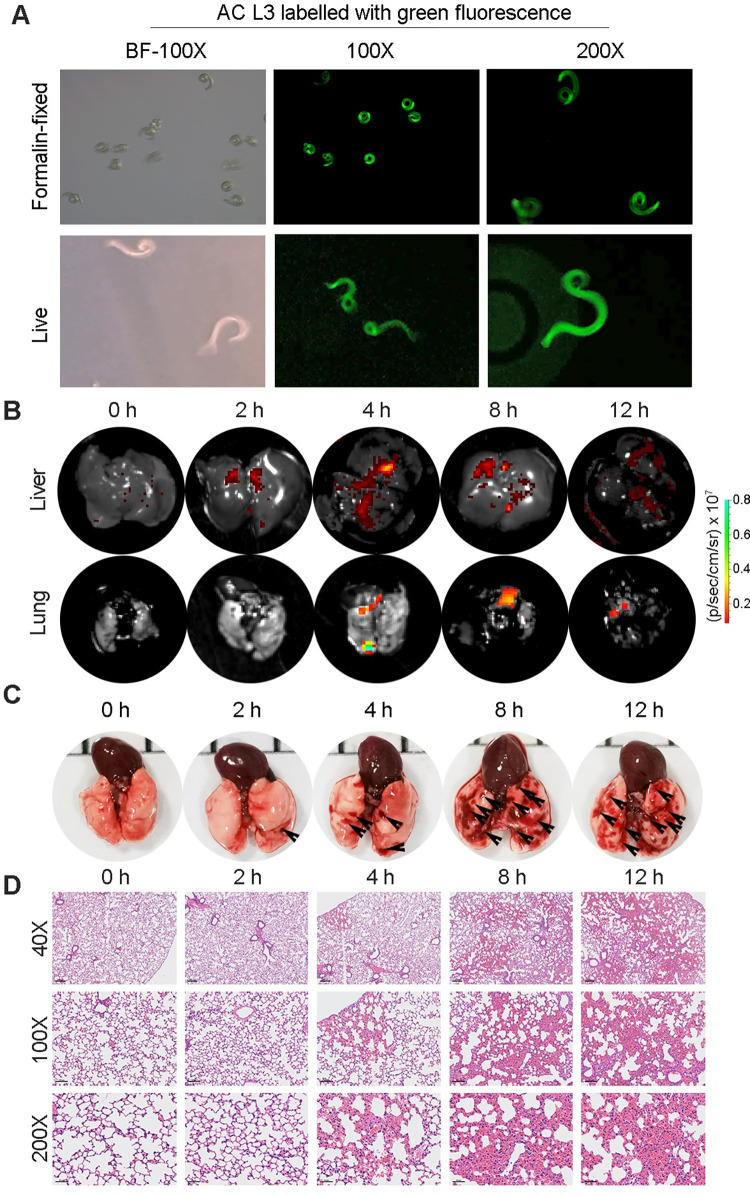
AC L3 migration through the mouse lung leads to pulmonary injury during the acute infection phase. (**A**) Representative pictures of formalin-fixed and live AC L3 labeled with green fluorescence to track the migration route in the host (n = 3). **(B)** Representative green fluorescence living images of mouse lungs (upper panel) and livers (lower panel) at 0, 2, 4, 8, and 12 hours after AC L3 infection (n = 3). **(C)** The gross morphology of mouse lungs at the indicated times after AC L3 infection (n = 5). The hemorrhagic lesions were shown by black arrows. **(D)** Pathologic configuration of mouse lungs during the acute infection phase, as displayed by H&E staining (n = 3). Magnifications: 40×, 100× and 200×.

Parasites usually parasitize the host in a tissue-specific manner. It is well known that AC will eventually parasitize the brain of nonpermissive hosts but the lungs of permissive hosts after infection [27]. During the process of migration from the original infection site to the final foci, the migration route of AC in permissive hosts, such as rats, is well documented, while that in nonpermissive hosts, including mice and humans, remains to be fully elucidated. To explore the migration route in a nonpermissive host, we first tried to label AC L3 with a green fluorescent dye. Under a fluorescence microscope, we confirmed that AC L3 cells were stably stained with green fluorescence under either formalin-fixed or live conditions (Fig 1A). Furthermore, AC L3 worms labeled with green fluorescence were found to retain excellent viability (S1 Video), and then these worms were perfused into the stomach of mice to establish an infection model. As reported, in permissive hosts such as rats, AC L3 migrates from the liver to the lung and then the brain during the acute infection phase (within 12 hours post-infection) [6]. To determine whether the same migration route also exists in nonpermissive hosts, such as mice, we monitored the fluorescence of the mouse liver and lung at 2, 4, 8 and 12 hours post-infection (collectively hpi) through live imaging. We found that the green fluorescence in the liver was detected at 2 hpi, peaked at 4 hpi and decreased at 8 hpi (Fig 1B upper panel), while that of the lung was detected at 4 hpi, peaked at 8 hpi and decreased at 12 hpi (Fig 1B lower panel). We further cut the mouse liver and lung tissues into pieces and counted the number of recovered AC worms under a stereomicroscope (S2 and S3 Videos). The results indicated that AC L3 reached the mouse liver and lung at 2 hpi and the brain at 8 hpi (S4 Table). Of note, the number of AC L3 present in the liver and lung peaked at 4 and 8 hpi, respectively (S4 Table). Overall, the results of live imaging and worm counts were consistent, which implied that AC L3 migrated from the liver to the lung and finally to the brain of the nonpermissive host mice.

The parasite migration process in the host leads to pathological injury, including hemorrhage and inflammatory responses [28]. We hence aimed to ask whether the AC L3 migration route resulted in tissue damage to the mouse liver and lung. For this purpose, we first observed morphological changes in the mouse liver during the acute infection phase of AC, but no significant morphological differences were seen (S1A Fig). Further pathological morphology analysis after H&E staining of the mouse liver gave the same results (S1B Fig). We next investigated the morphological and pathological changes in the mouse lung during the acute infection phase. Remarkably, AC L3 migration caused progressively more severe lung hemorrhage from 2 to 12 hpi (Fig 1C), and H&E staining yielded identical results (Fig 1D). Moreover, we observed significant alveolar wall thickening, inflammatory cell infiltration, and alveolar congestion (Fig 1D). Combined with the symptom of shortness of breath, we concluded that AC L3 migrates from the mouse liver to the lung but specifically leads to pulmonary injury (pneumonia) during the acute infection phase.

### Persistent pneumonia in permissive and nonpermissive hosts during the early stage of AC infection

**Fig 2:**
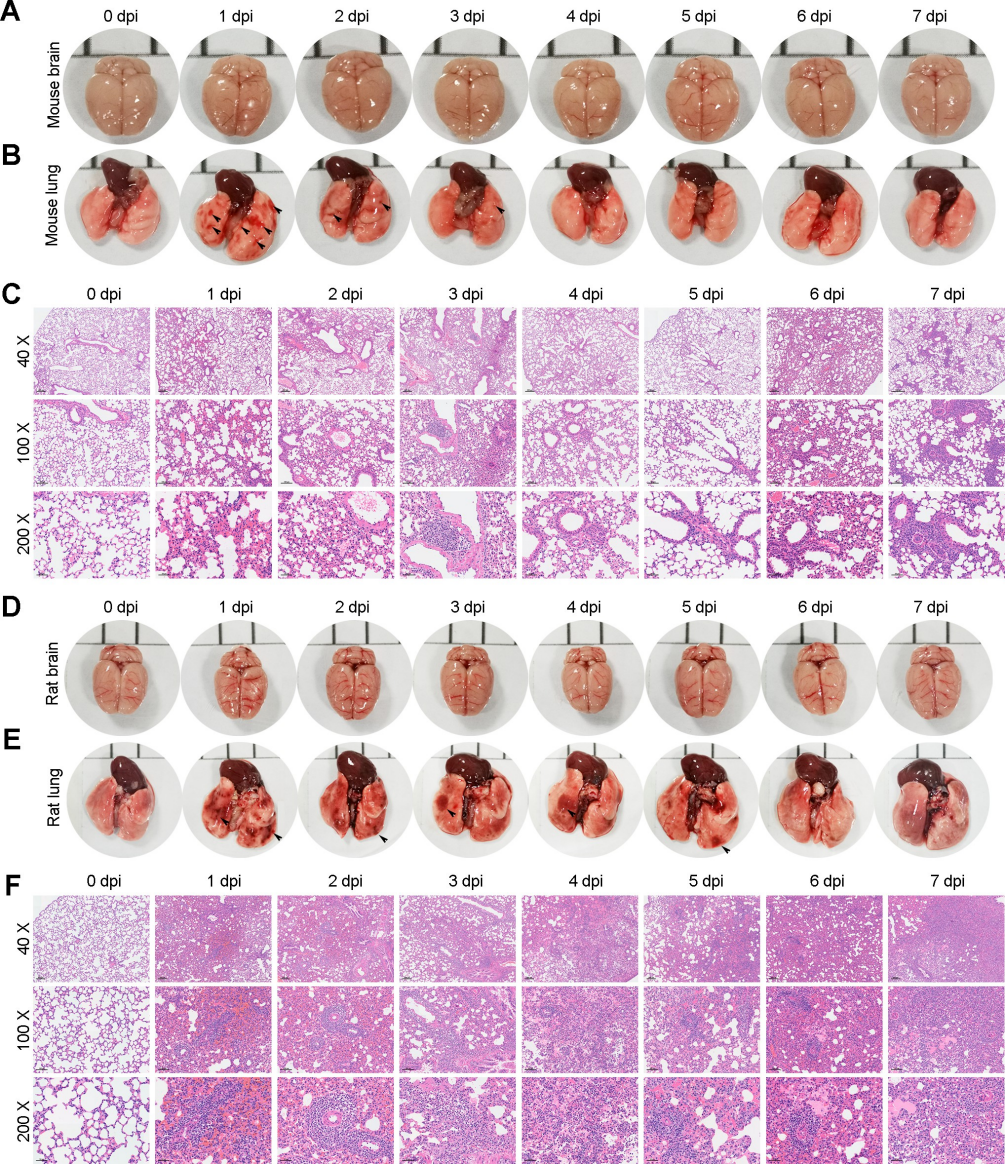
Persistent lung injury in permissive and nonpermissive hosts during the early stage of AC infection. **(A-B)** Representative gross morphology pictures of mouse brains (A) and lungs (B) at 1, 2, 3, 4, 5, 6 and 7 days post-infection (dpi) of AC (n = 5). The hemorrhagic lesions were shown by black arrows. **(C)** H&E staining shows the pathological injury of the mouse lung at the indicated time of AC infection (n = 3). **(D-E)** Representative images of rat brains (D) and lungs (E) at 1, 2, 3, 4, 5, 6, and 7 dpi of AC (n = 5). The hemorrhagic lesions were shown by black arrows. **(F)** H&E staining shows the pathological injury of rat lungs at the indicated time of AC infection (n = 3). Magnifications: 40×, 100× and 200×.

After the acute infection phase (within 12 hours), AC L3 left the mouse liver and lung, and the infection process entered the early phase (from 1 to 7 days post-infection) when AC L3 completely migrated to the mouse brain. To explore how AC worms influenced the mouse brain and lung during this phase, we first examined the morphological alterations at the indicated numbers of days post-infection (collectively dpi) and found no obvious bleeding spots or thickening of meninges in the mouse brain (Figs 2A and S2A). However, in the mouse lung, we observed significant and multiple bleeding spots at 1 dpi, which decreased from 1 to 3 dpi (Fig 2B) and disappeared at 4 dpi, implying that AC L3 completed migration from the lung to the brain at 4 dpi. Since we did not observe any bleeding spots in the mouse brain and other studies have documented that AC worms do not cause notable inflammation in the brain before 7 dpi, we focused on lung injury during this phase. Further H&E staining analysis of mouse lungs suggested that hemorrhage dominated at 1 and 2 dpi, while inflammatory cell infiltration and alveolar wall thickening were the main pathological alterations from 3 to 7 dpi (Fig 2C), which was consistent with the morphological changes. Furthermore, we also investigated the effect of AC worms on the brains and lungs of permissive host rats during the early infection phase. No obvious morphological alterations were observed in the rat brain (Figs 2D and S2B). Compared with rat lungs at 0 dpi, striking bleeding spots were seen at 1 dpi, which decreased from 2 to 7 dpi but were persistent (Fig 2E). The pathological configuration of the rat lung showed sustained hemorrhage, thickening of the alveolar wall and inflammatory cell infiltration (Fig 2F). Therefore, AC led to persistent pneumonia of permissive and nonpermissive hosts during the early infection phase.

### Pneumonia exhibited a Th2 bias during the early infection phase

**Fig 3:**
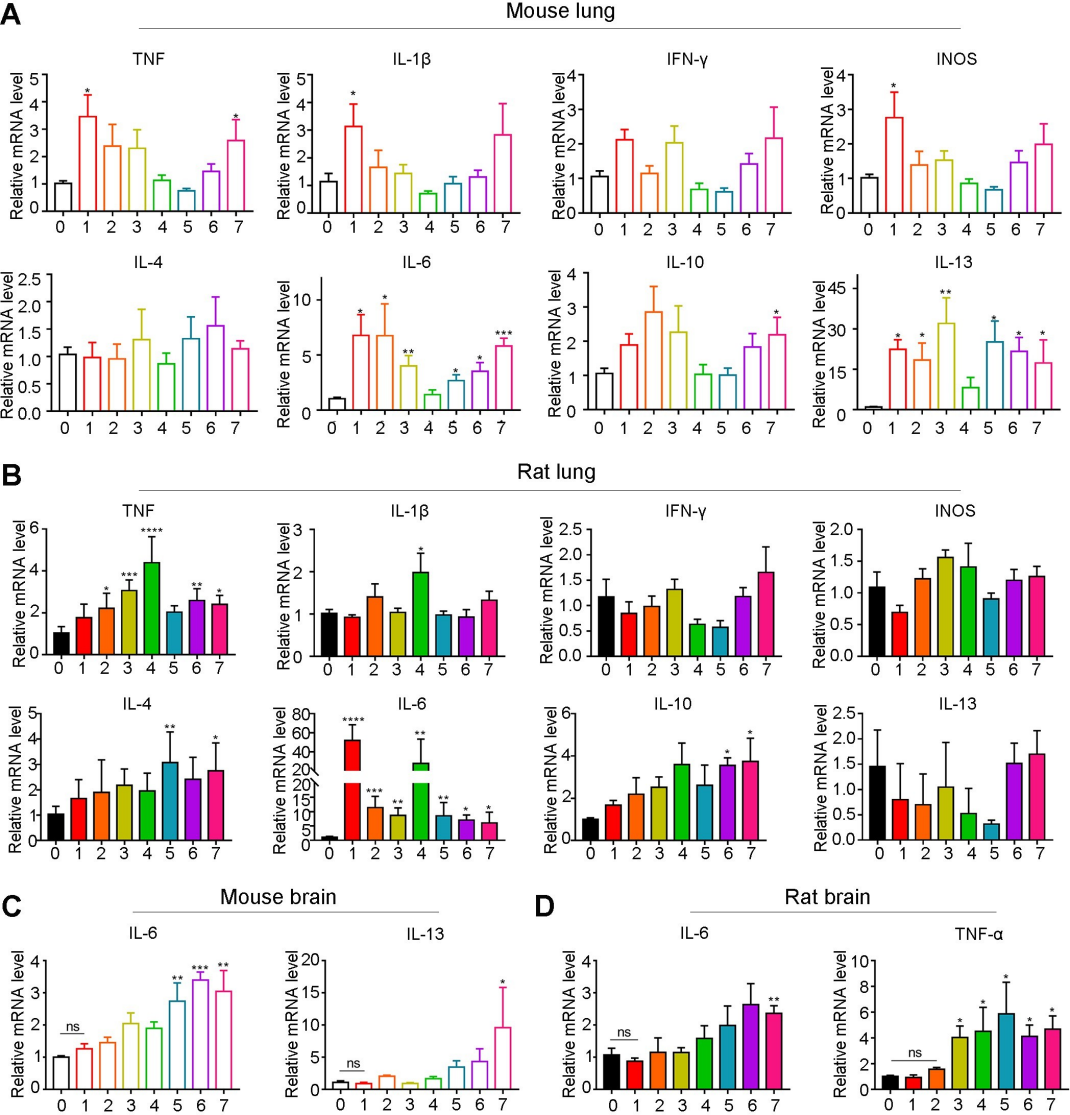
Th1 and Th2 cytokines are significantly upregulated during the early stage of AC infection. **(A-B)** The relative mRNA levels of Th1 cytokines (TNF-α, IL-1β, IFN-γ, INOS) and Th2 cytokines (IL-4, IL-6, IL-10, IL-13) in mouse (A) and rat (B) lungs at 1, 2, 3, 4, 5, 6, and 7 dpi of AC (n = 4). **(C-D)** The relative mRNA levels of IL-6 and IL-13 in mouse (C) and rat (D) brains at the indicated times after AC infection (n = 4). *p < 0.05, **p < 0.01, ***p < 0.001, ****p < 0.0001 compared to 0 dpi.

Next, we aimed to pinpoint how AC affected host pneumonia during the early infection phase. To this end, we detected the expression of Th1 (TNF-α, IL-1β, IFN-γ, INOS) and Th2 cytokines (IL-4, IL-6, IL-10, IL-13) that were reported to be involved in parasitic immunity in mouse and rat lungs. The results in mouse lungs indicated that the levels of Th1 cytokines (TNF-α, IL-1β and INOS) were significantly elevated by approximately 3-fold at 1 dpi compared with 0 dpi but gradually decreased to normal at 2–4 dpi and slightly increased again from 5–7 dpi (Fig 3A upper). IFN-γ levels also showed a slight increase, but this change was not significant (Fig 3A upper). For Th2 cytokines, we observed that levels of IL-6 strikingly increased by 7-fold and the levels of IL-13 increased by 20-fold (Fig 3A lower) at 1 dpi compared to 0 dpi and dropped to normal at 4 dpi but rose again to high levels from 5–7 dpi. However, IL-4 and IL-10 levels were only marginally changed (Fig 3A lower). In contrast, in rat lungs, we found that none of the Th1 cytokines were elevated at 1 dpi. Only TNF progressively increased from 2 to 4 dpi and peaked at 4 dpi but decreased again from 5 to 7 dpi (Fig 3B upper). For Th2 cytokines, IL-6 levels were notably upregulated by 50-fold at 1 dpi compared with 0 dpi and maintained at 5-fold above from 2 to 7 dpi (Fig 3B lower). IL-4 and IL-6 levels were gradually elevated and reached 2-fold above at 7 dpi (Fig 3B lower). IL-13 levels were not changed from 1 to 7 dpi (Fig 3B lower). Furthermore, we also detected the expression level of most significantly upregulated cytokines implicated in host pneumonia in the host brain and found that none of them was upregulated at 1 dpi, but there was a gradual increase from 2 to 7 dpi (Fig 3C and 3D). Overall, during the early infection phase, AC migration led to persistent pneumonia characterized by a Th2 bias in mice (mainly IL-13 and IL-6) and rats (mainly IL-6).

### High-dose AC infection leads to fatal pneumonia in nonpermissive hosts during the early infection phase

**Fig 4:**
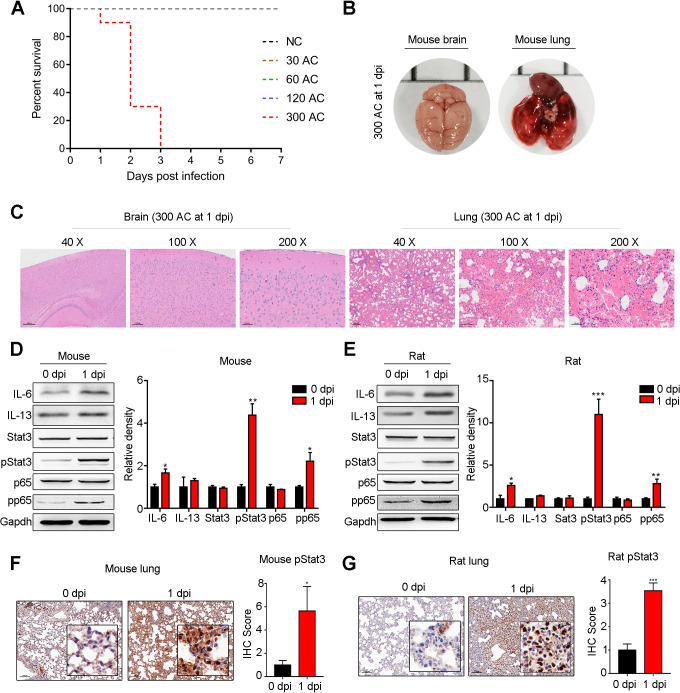
High-dose AC infection leads to fatal lung injury in nonpermissive hosts during the early infection phase. **(A)** Survivals of mice infected with different doses of AC L3 were evaluated by Kaplan–Meier curve analysis (n = 10). NC, negative control. **(B-C)** Appearance (B) and H&E staining (C) of mouse brain and lung at 1 dpi of 300 AC L3 (n = 3). **(D-E)** Immunoblotting analysis of IL6, IL13, Stat3, phospho-Stat3 (pStat3), NF-κB p65 (p65), phospho-NF-κB p65 (pp65) and Gapdh in mouse (D, left) and rat (E, left) lungs at 1 dpi AC (n = 3). The intensity of the indicated proteins was compared with a histogram between 1 dpi group and 0 dpi group for mouse (D, right) and rat (E, right) lungs. **(F-G)** Representative images of pStat3 IHC staining (left panel) and the corresponding IHC (right panel) score displaying the pathological configuration of mouse (F) and rat (G) lungs at 0 and 1 dpi of AC (n = 3). *p < 0.05, **p < 0.01, ***p < 0.001 compared to 0 dpi.

As AC induced a notable inflammatory response and injury in the host lung during the acute and early infection phases, we next asked whether this alteration would cause host death. For this purpose, mice were separately infected with different doses of AC L3 (30, 60, 120, 300), and survival curves were generated during the early infection phase (1–7 dpi). The results showed that no mouse died after infection with 30, 60 and 120 AC L3, while 10% of mice infected with 300 AC L3 died at 1 dpi, 70% died at 2 dpi and all died at 3 dpi (Fig 4A). To pinpoint the cause of death, the brain and lung tissues of mice that died at 1 dpi were removed and photographed, followed by H&E staining. As presented, no significant bleeding or tissue injury occurred in the mouse brain (Fig 4B left), while in the mouse lung, we observed areas of severe bleeding (Fig 4B right). Consistently, H&E staining of the mouse brain showed a normal tissue structure (Fig 4C left), but that of the mouse lung revealed very severe alveolar congestion and inflammatory cell infiltration (Fig 4C right). As IL-6 and IL-13 were the main cytokines upregulated in mouse lungs during the early infection phase, we aimed to detect the protein levels of these two cytokines in the lungs of mice that died at 1 dpi and found that the protein level of IL-6 was significantly elevated but that of IL-13 showed only a slight increase (Fig 4D). Protein–protein interaction analysis indicated that the transcription factors STAT3 and NF-κB play a central role in regulating Th1 and Th2 cytokine expression (S3 Fig). We thus evaluated the activation of Stat3 and p65 in the mouse lung. As shown, the protein levels of total Stat3 and p65 exhibited no obvious change upon AC infection at 1 dpi, while those of activated Stat3 and p65 were significantly upregulated by 4- and 2-fold, respectively (Fig 4D). Consistently, we observed similar results in rat lungs, with 10-fold and 2-fold upregulation of activated Stat3 and p65, respectively (Fig 4E). These data indicated that activation of Stat3 played more important roles in the host lung at 1 dpi. In addition, we further substantiated the evident activation of Stat3 by immunohistochemistry in mouse (Fig 4F) and rat (Fig 4G) lungs at 1 dpi. Altogether, the results suggested that infection with a high dose of AC L3 could lead to fatal pneumonia in a nonpermissive host during the early infection phase before the manifestation of eosinophilic meningitis.

### Sustained interstitial pneumonia in permissive and nonpermissive hosts during the late AC infection phase

**Fig 5:**
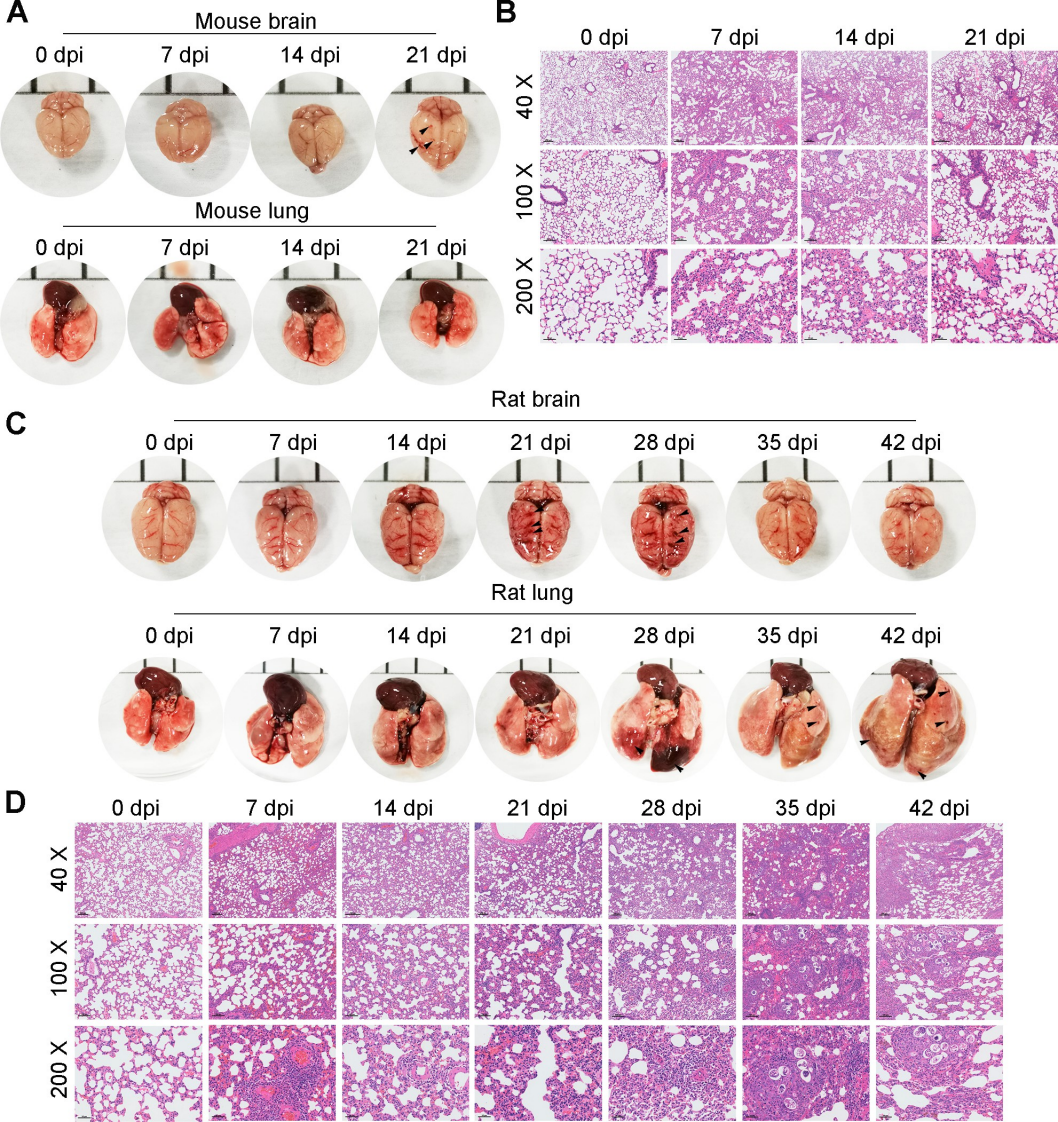
Sustained lung injury in permissive and nonpermissive hosts during the late stage of AC infection. **(A)** Representative images of mouse brains and lungs at 0, 7, 14, and 21 dpi of AC (n = 5). **(B)** H&E staining displays the pathological configuration of mouse lungs (n = 3). **(C)** Representative pictures of rat brains and lungs at 0, 7, 14, 21, 28, 35, and 42 dpi of AC (n = 5). **(D)** H&E staining displays the pathological configuration of rat lungs (n = 3). Magnifications: 40×, 100× and 200×.

Based on laboratory observations, nonpermissive host mice infected with 30 AC L3 exhibited severe eosinophilic meningitis at 21 dpi and died before 28 dpi. In contrast, permissive host rats infected with 100 AC L3 survived more than 42 days. To further explore how AC exerted influences on the host lung and brain during the late infection phase (7 to 21 dpi for mice and 7–42 dpi for rats), mice and rats were infected with 30 and 100 AC L3, respectively, followed by morphological and pathological observation of the brain and lung on the indicated numbers of days post-infection. We found that AC caused notable hemorrhage and thickening meninges in the mouse brain surface at 21 dpi (Figs 5A upper and S4A) but no visible hemorrhage in mouse lung during the late infection phase (Fig 5A lower). Considering that the pathological alteration of the mouse brain caused by AC in the late infection phase has been well documented, we focused on pathological changes in the mouse lung. Of note, although there was no grossly visible hemorrhage during the late infection phase, H&E staining revealed sustained slight interstitial pneumonia characterized by a thickened alveolar wall and inflammatory cell infiltration (Fig 5B). For permissive host rats, significant bleeding areas and thickening meninges in the brain were visible only at 21 and 28 dpi (Figs 5C upper and S4C) and those of the lung were visible only at 28, 35, and 42 dpi (Fig 5C lower), which was in line with the fact that AC started to migrate from the rat brain to the lung at 28 dpi. Configuration analysis of rat lungs indicated that AC infection led to persistent alveolar wall thickening and inflammatory cell infiltration during the late infection phase (Fig 5D), although no visible bleeding areas were observed at 7, 14 and 21 dpi. Taken together, these results suggested that sustained interstitial pneumonia occurred in permissive and nonpermissive hosts during the late AC infection phase.

### Persistently enhanced Th2 cytokine expression and Stat3 and NF-κB signaling during the late infection phase

**Fig 6:**
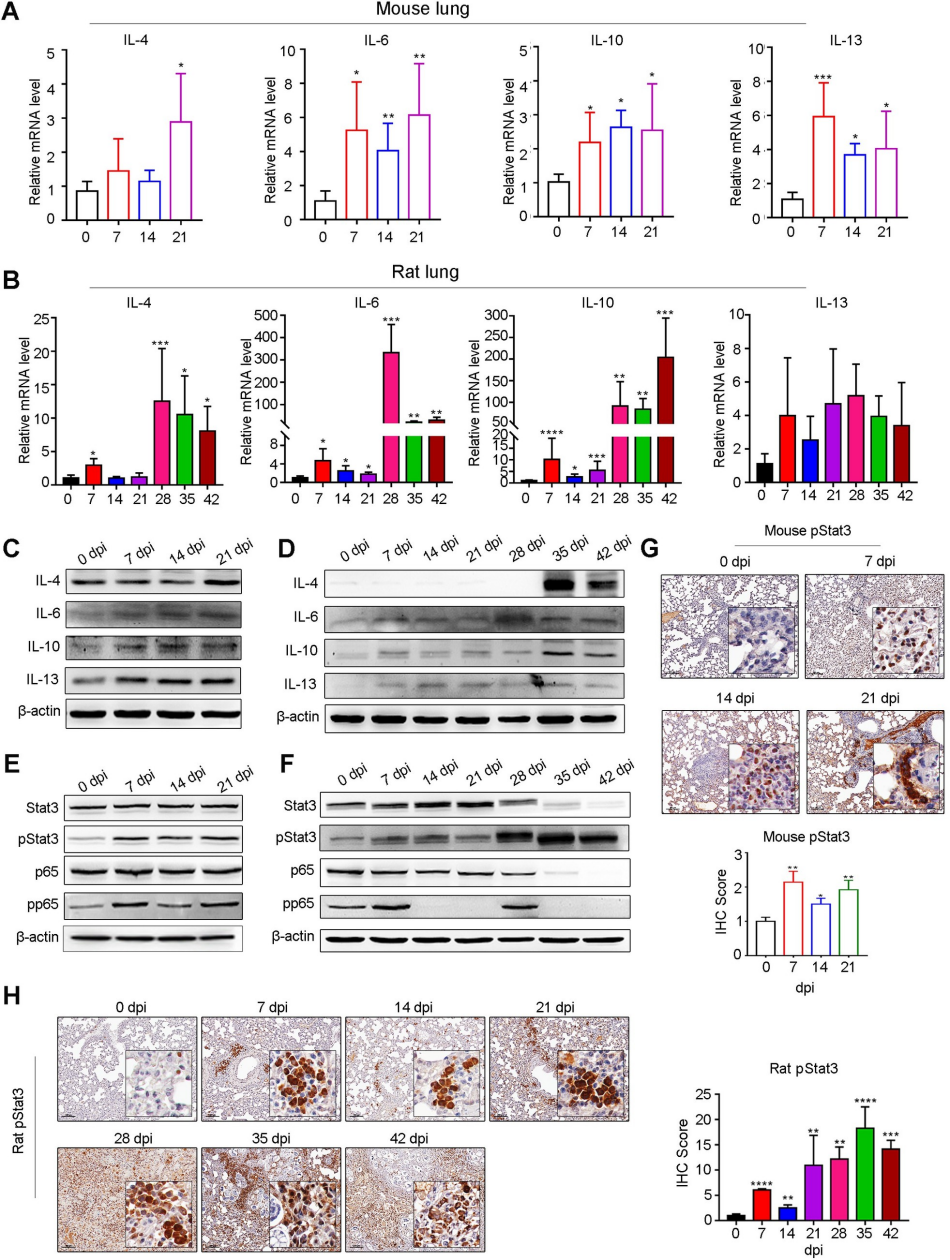
Persistently enhanced Th2 cytokine expression and Stat3 and NF-κB signaling during the late infection phase. **(A-B)** The relative mRNA levels of Th2 cytokines (IL-4, IL-6, IL-10, IL-13) in mouse (A, at 0, 7, 14, 21 dpi of AC) and rat (B, at 0, 7, 14, 21, 28, 35, 42 dpi of AC) lungs were determined by RT–qPCR (n = 4). **(C-D)** The protein levels of Th2 cytokines (IL-4, IL-6, IL-10, IL-13) in mouse (C) and rat lungs(D) were determined by immunoblotting (n = 3). **(E-F)** The translational levels of Stat3 signaling (Stat3, pStat3) and NF-κB signaling (p65, pp65) in mouse(E) and rat(F) lungs were assessed by immunoblotting (n = 3). **(G-H)** The protein level of pStat3 in mouse (G, at 0, 7, 14, 21 dpi of AC) and rat (H, at 0, 7, 14, 21, 28, 35, 42 dpi of AC) lungs was tested by IHC (n = 3). *p < 0.05, **p < 0.01, ***p < 0.001, ****p < 0.0001 compared to 0 dpi.

Next, we aimed to detect the expression of Th1 and Th2 cytokines in mouse and rat lungs during the late infection phase. Among Th1 cytokines in the mouse lung, the level of TNF-α was significantly elevated by less than 2-fold (S5A Fig) and that of IL-1β was elevated by 2-fold at 21 dpi, while INOS and IFN-γ exhibited only a slight increase (S5A Fig). Among Th2 cytokines in the mouse lung, we found that the levels of all four marker cytokines (IL-4, IL-6, IL-10, and IL-13) progressively increased with infection time (Fig 6A); similarly, the levels of IL-6, IL-10 and IL-13 were upregulated in the mouse brain (S4B Fig). However, in rat lungs, only TNF-α of Th1 cytokines was persistently increased during the late infection phase (S5B Fig). IL-1β and INOS exhibited a sudden rise at 28 dpi but quickly dropped down to normal levels (S5B Fig). In contrast, the Th2 cytokines IL-4 and IL-6 cytokines displayed a striking increase at 28 dpi and decreased to a relatively low level later but were still higher than normal levels (Fig 6B). Of note, IL-10 exhibited progressively enhanced levels with increasing infection time (Fig 6B). In rat brain, the levels of IL-4 were significantly decreased, IL-10 progressively increased before 28 dpi but dropped to normal level at 35 and 42 dpi, IL-6 and IL-13 showed no changes (S4B Fig). Overall, Th2 cytokines dominated the inflammation of the lung during the late infection phase in both permissive and nonpermissive hosts of AC. Furthermore, at the protein level, we also confirmed that all four Th2 cytokines (IL-4, IL-6, IL-10, and IL-13) were progressively enhanced in both mouse (Figs 6C and S6A) and rat lungs during the late infection phase of AC (Figs 6D and S6B). Immunoblot analysis suggested that Stat3 and p65 were notably and sustained activated in mouse lungs during the late infection phase (Figs 6E and S6A). In contrast, in rat lungs, Stat3 was progressively activated (Figs 6F and S6B), while activation of p65 occurred only at 7 and 28 dpi (Figs 6F and S6B). Further IHC results also revealed the significant progressive activation of Stat3 in both mouse (Fig 6G) and rat (Fig 6H) lungs during the late infection phase of AC.

### Innate immune myeloid cells dominated persistent but nonfatal interstitial pneumonia during the late infection phase

**Fig 7:**
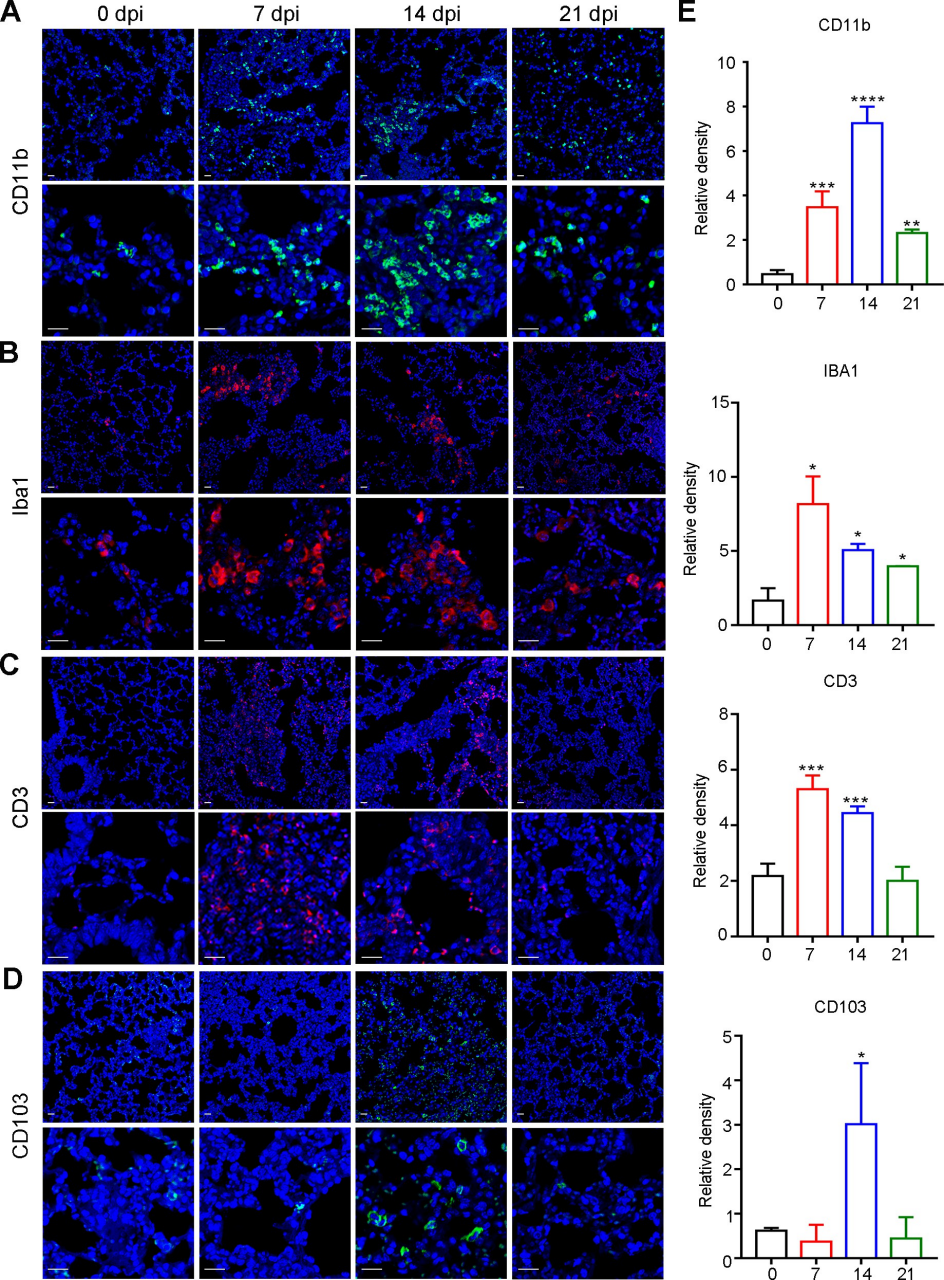
The inflamed immune microenvironment in mouse lungs during the late stage of AC infection. **(A-D)** Representative immunofluorescence images of myeloid cells (A, CD11b as the specific marker), macrophages (B, IBA1 as the specific marker), T cells (C, CD3 as the specific marker) and dendritic cells (D, CD103 as the specific marker) in mouse lungs at 0, 7, 14, 21 dpi of AC (n = 3). **(E)** Quantitative analysis of the indicated markers for immunofluorescence in a-d. scale bar: 50μm. *p < 0.05, **p < 0.01, ***p < 0.001, ****p < 0.0001 compared to 0 dpi.

**Fig 8:**
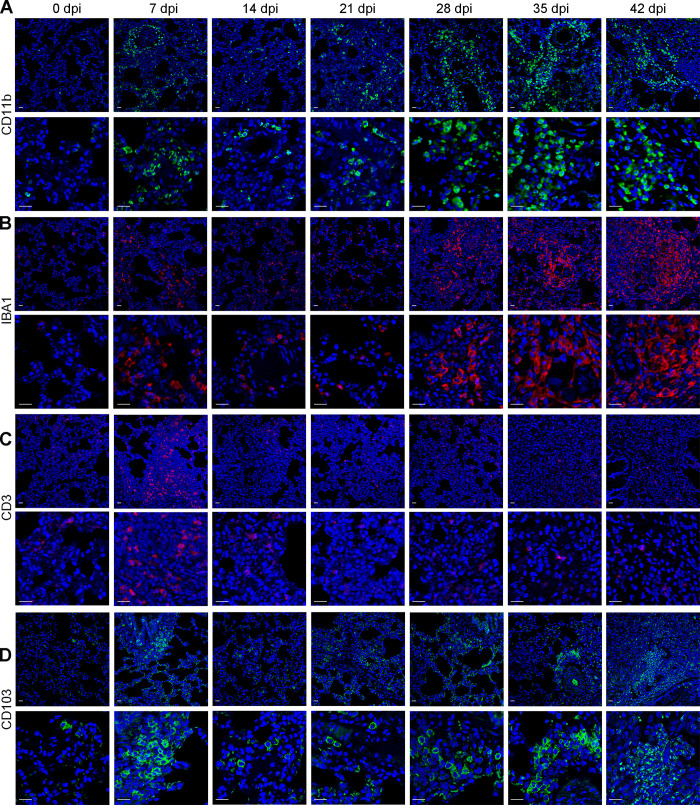
The inflamed immune microenvironment in rat lungs during the late stage of AC infection. **(A-D)** Representative immunofluorescence images of myeloid cells (A, CD11b as the specific marker), macrophages (B, IBA1 as the specific marker), T cells (C, CD3 as the specific marker) and dendritic cells (D, CD103 as the specific marker) in rat lungs at 0, 7, 14, 21, 28, 35, and 42 dpi of AC (n = 3). scale bar: 50μm.

As inflammatory cells were present in the lungs of the host (Fig 5B and 5D), we next proceeded to distinguish the specific immune cell types implicated in interstitial pneumonia during the late infection phase of AC. Here, we selected CD11b and IBA1 to reflect innate immune cell enrichment, CD3 to represent adaptive immune cells and CD103 to represent antigen-presenting cells (dendritic cells). In the mouse lung, at the transcriptional level, we found that CD11b levels were persistently upregulated during the late infection phase, while those of IBA1, CD3 and CD103 were remarkably increased at 7 and 14 dpi but dropped down to normal levels at 21 dpi (S7A Fig). Immunofluorescence results showed that CD11b^+^ and IBA1^+^ innate immune cells exhibited sustained enrichment during the late infection phase (Fig 7A, 7B and 7E), CD3^+^ adaptive immune cells (T cells) were significantly aggregated at 7 and 14 dpi but returned to basal levels (Fig 7C–7E), and CD103^+^ antigen-presenting cells (dendritic cells) were notably enriched only at 14 dpi (Fig 7D and 7E). In the rat lung, at the transcriptional level, we found that CD11b, IBA1 and CD103 levels were all elevated during the late infection phase, but CD3 levels were enhanced only at 7 dpi (S7B Fig). Further immunofluorescence results indicated that CD11b^+^, IBA1^+^ and CD103^+^ immune cells were all significantly enriched during the late infection phase (Figs 8A, 8B, 8D and S7C), while CD3^+^ immune cells were aggregated only at 7 dpi (Figs 8C and S7C), which was consistent with the transcriptional results. Altogether, these data suggested that innate immune myeloid cells were implicated in persistent host interstitial pneumonia throughout the late infection phase.

Next, to investigate whether sustained interstitial pneumonia could be fatal during the late infection phase, we evaluated the cell death of mouse lung cells and found that the expression of critical genes involved in cell death (RIP3, RIP1, Caspase-3, Caspase-8) exhibited no change (S8A Fig). Further flow cytometry results indicated that the percentage of dead mouse lung cells between 0 dpi and 7, 14, and 21 dpi showed no significant change (S8B Fig). Similarly, in rat lungs, we observed the same phenotype (S8C and S8D Fig). Taken together, our data suggested that interstitial pneumonia was persistent but nonfatal during the late infection phase of AC.

### AC induced fibrosis in rat lungs but not mouse lungs during the late infection phase

**Fig 9:**
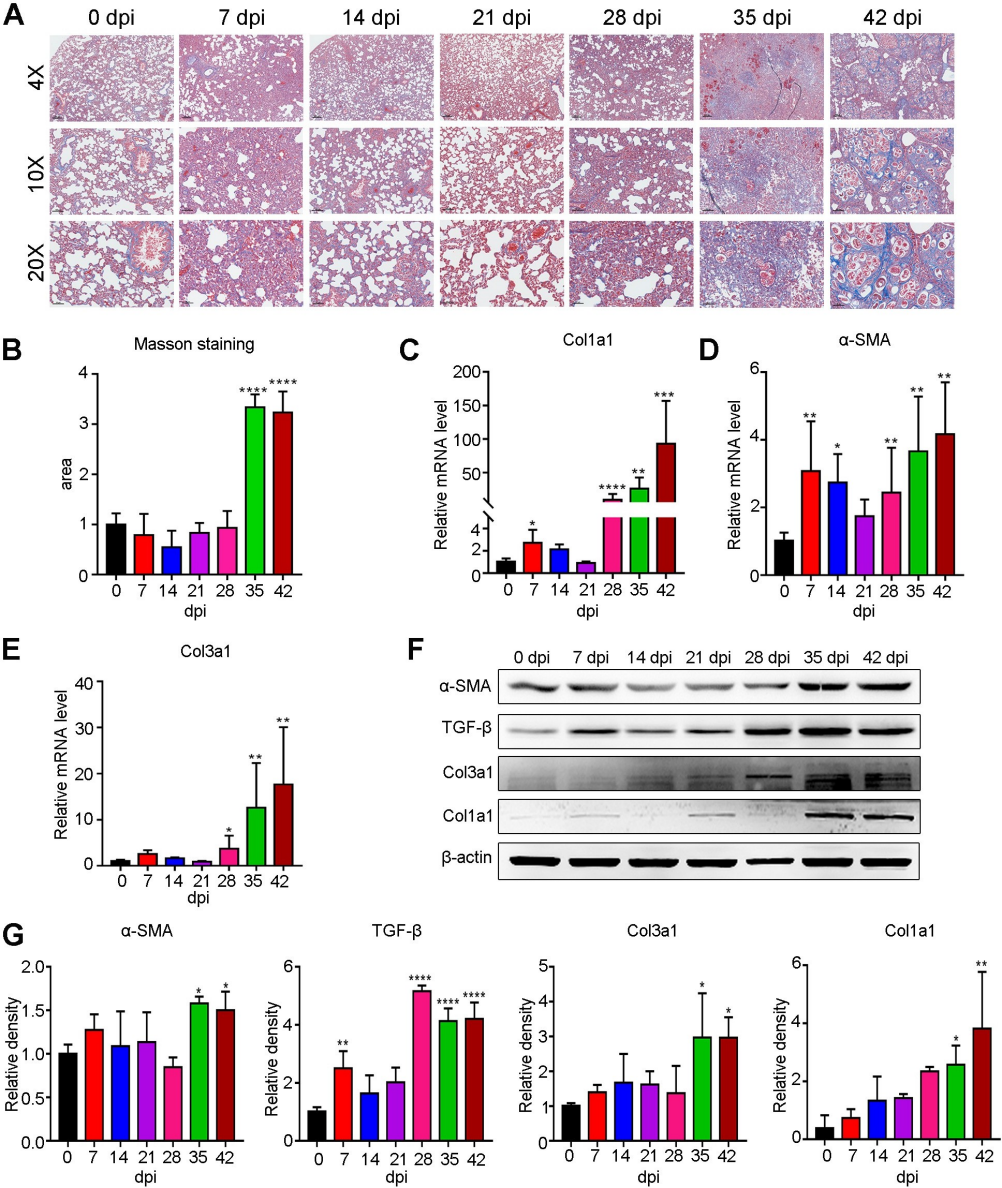
Persistent inflammation leads to notable fibrotic lesions in rat lungs during the late stage of AC infection. **(A)** Representative Masson staining pictures showing the fibrotic lesions of rat lungs at the indicated dpi of AC (n = 3). **(B)** Quantitative analysis of Masson staining in A. **(C-E)** The transcriptional level of fibrosis-related genes (Col1a1, Col3a1 and α-SMA) in rat lungs at the indicated dpi of AC (n = 4). **(F)** The protein levels of fibrosis-related genes (α-SMA, TGF-β, Col1a1 and Col3a1) were significantly upregulated in rat lungs during the late stage of AC infection, as shown by immunoblotting (n = 3). **(G)** Differences in the relative protein intensity between groups were compared with the histogram. *p < 0.05, **p < 0.01, ***p < 0.001, ****p < 0.0001 compared to 0 dpi.

Persistent inflammation can trigger collagen deposition, which leads to fibrosis [29]. To further investigate whether AC infection-induced lung inflammation leads to pulmonary fibrosis, we performed Masson staining, RT–qPCR and immunoblot analysis and found no obvious fibrosis in the mouse lung (S9A and S9B Fig), which was further confirmed by the lack of elevated transcript and protein levels of Col1a1, Col3a1, α-SMA and TGF-β (S9C–S9E Fig). In contrast, we observed prominently enhanced fibrosis (approximately 3-fold) in rat lungs at 35 and 42 dpi, as revealed by Masson staining (Fig 9A and 9B). Consistently, the mRNA (Fig 9C–9E) and protein (Fig 9F and 9G) levels of Col1a1, Col3a1 and α-SMA were upregulated from 28 to 35 dpi. TGF-β, the critical regulator of fibrosis, was also markedly activated (Fig 9F–9G). Overall, AC infection-induced sustained lung inflammation and could lead to fibrosis in rat but not mouse lungs during the late infection phase.

### Blockade of Stat3/IL-6 signaling attenuates AC infection-induced pulmonary inflammation

**Fig 10:**
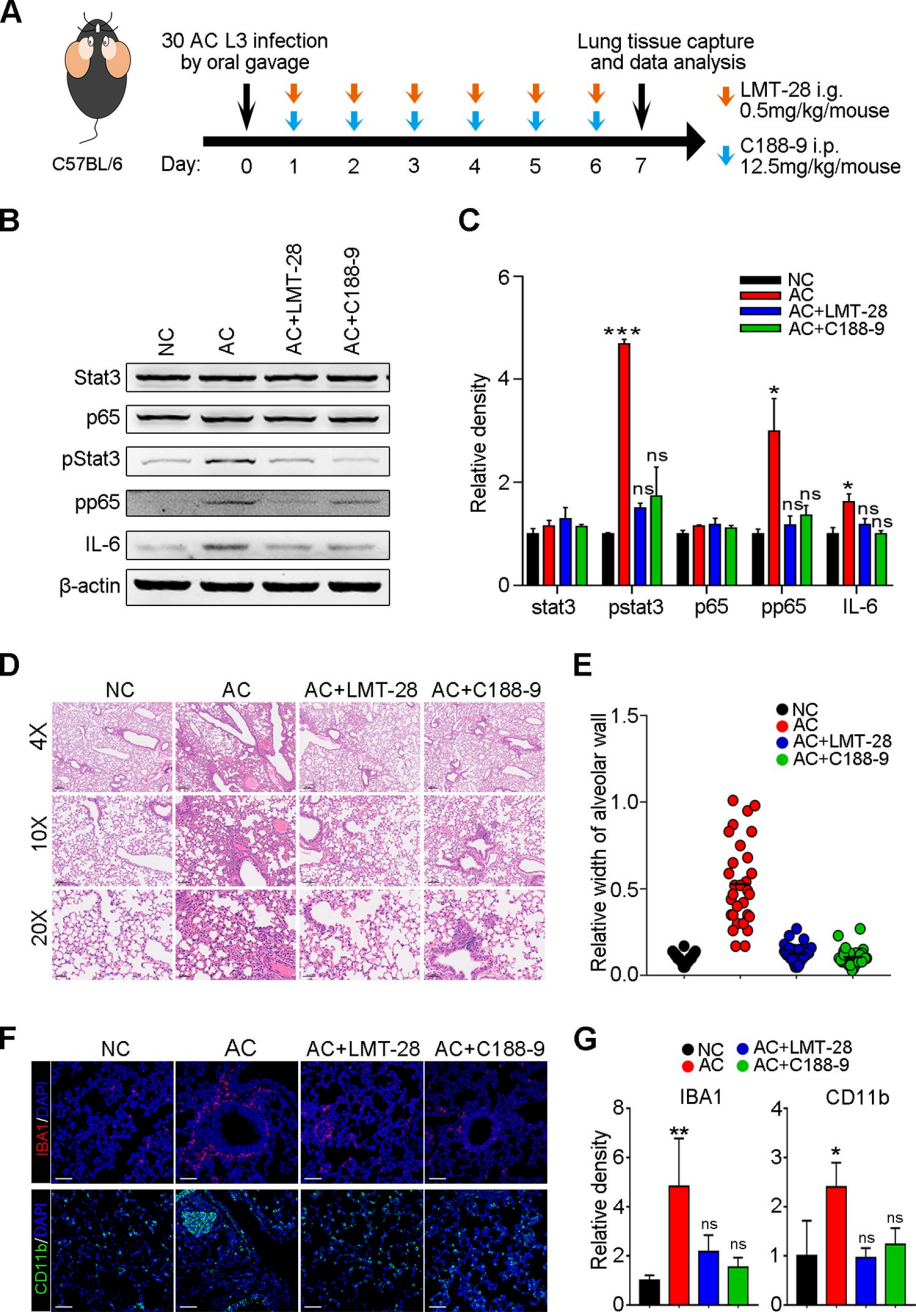
Blockade of Stat3/IL-6 signaling attenuates AC infection-induced inflammation and pathological damage in the host lung. (**A**) The flowchart of animal experiment. Mice were infected with 30 AC L3 followed by LMT-28 (oral gavage) and C188-9 (intraperitoneal injection) treatment from 1 to 6 dpi. At 7 dpi, mice were sacrificed, and lung tissues were captured for further investigation. (**B**) The protein levels of Stat3/IL-6 signaling and NF-κB p65 were determined by Western blot (n = 3). (**C**) Relative protein intensity in B was quantified. (**D**) H&E staining displays the pathological configuration of mouse lungs (n = 3). (**E**) Relative width of the alveolar wall of mouse lungs in (D). (**F**) Representative immunofluorescence images of myeloid cells and macrophages in mouse lungs (n = 3). (**G**) The relative immunofluorescence density of CD11b and IBA1 in mouse lungs. Magnifications: 40×, 100× and 200×.

Our data indicated that AC infection could induce fatal pneumonia in nonpermissive host mice during the acute and early infection phases (Fig 4A). Fatal pneumonia was attributed to severe alveolar congestion and the inflammatory response in the mouse lung (Fig 4B and 4C). Importantly, Stat3/IL-6 signaling was dominantly enhanced at 1 dpi (Fig 4D–4G). Thus, we asked whether intervention with Stat3/IL-6 signaling inhibitors could blunt pneumonia. To this end, mice infected with 30 AC L3 cells were separately treated with a Stat3 inhibitor (C188-9) and an IL-6 inhibitor (LMT-28) from 1 to 6 dpi, and inflammation and pathological damage to the lung were evaluated at 7 dpi (Fig 10A). As expected, the upregulation of Stat3 signaling and IL-6 caused by AC infection was significantly attenuated (Fig 10B and 10C). Notably, the activation of NF-κB p65 was also mitigated (Fig 10B and 10C). Pathological analysis of the morphology of the mouse lung revealed that AC infection caused alveolar edema, and inflammatory cell infiltration was also diminished upon LMT-28 and C188-9 treatment (Fig 10D). The thickening of alveolar walls was also abated (Fig 10E). In addition, the enrichment of innate immune myeloid cells caused by AC was notably counteracted (Fig 10F and 10G) by LMT-28 and C188-9. Overall, we confirmed that pneumonia in the host was mediated by the Stat3/IL-6 pathway and that blockade of Stat3/IL-6 signaling could significantly attenuate AC infection-induced pneumonia in the host.

## Discussion

As it is well known that humans and mice infected by AC develop eosinophilic meningitis, an increasing number of studies in recent decades have focused on characterizing and searching for therapeutic strategies for AC-induced meningitis [30]. We should pay the most attention to severe eosinophilic meningitis, which can cause death and permanent irreversible nerve damage [31], but this manifestation was observed only in the late phase of AC infection (21 dpi for mice). Of note, AC larvae migrate from the mouse intestinal wall to the brain, where they die and trigger fatal meningitis [32]; however, during the acute and early infection phases (before 7 dpi), when AC larvae pass through host organs, whether they can cause accompanying tissue damage is unclear, and the characteristics of these injuries remain elusive. In addition, the migration route of AC in permissive host rats is very clear, but that in nonpermissive hosts still needs to be fully elucidated. In this study, we first exploited live imaging technology to visualize the migration route of AC in mice and found that AC larvae migrated from the mouse intestinal wall to the liver at 2 hpi, from the liver to the lung at 4 hpi and then from the lung to the brain at 8 hpi. More importantly, we first reported that during the acute and early infection phases, AC larvae could induce mouse fatal pneumonia, with significant activation of Stat3/IL-6 signaling and enrichment of myeloid cells. Furthermore, we revealed that inhibitors targeting Stat3/IL-6 could notably attenuate severe pneumonia during the early infection phase.

AC was first discovered in rat lungs by Chen in 1935 [27], and then the migration route of AC in rats was revealed [12]. As reported, AC L3 migrated from the rat intestinal wall to the liver within 2 hpi, to the lung at 2 hpi and the brain at 8 hpi, and finally returned from the brain to the lung at 28 dpi [12]. Afterward, rats were confirmed as permissive hosts, but mice and humans were confirmed as nonpermissive hosts. To date, the migration of AC in nonpermissive host mice is thought to be similar to that in rats, but direct laboratory evidence is still lacking. Here, we first provide strong experimental evidence that AC L3 used the same migration route in mice as in rats, but the timeline was slightly different. AC migrated from the liver to the lung at 2 hpi in rats but at 4 hpi in mice, which might be caused by the faster blood flow rate in the rat liver and lung than in the mouse liver and lung [33]. In addition, previous studies suggested that pneumonia could be induced by a variety of parasites, such as *Strongyloides*, *Paragonimus*, and *Toxocara* [16], and these parasites cause pneumonia mainly through direct contact or systemic inflammation. As AC larvae migrate from the mouse lung and lead to a host immune response, we continued to explore whether AC can cause pneumonia. Our results confirmed that during the acute and early infection phases, AC larvae induced fatal pneumonia, while during late infection, AC induced sustained but slight interstitial pneumonia. The reason why AC caused different types of pneumonia during the early and late infection phases might be that during the acute infection phase, AC larvae caused direct mechanical injuries in the host lung, which led to alveolar congestion, but during late infection, AC was present only in the host brain and induced systemic cytokine-dependent interstitial pneumonia. In addition, we observed that although AC larvae passed through the liver and lung, they caused only lung injuries. It is likely that AC traveled in the circulating blood and entered the liver through inferior vena cava regurgitation to the hepatic vein but rarely arrived at the intrahepatic capillary. In contrast, when AC migrates through the lung in the circulating blood, it can easily damage pulmonary capillaries located in the alveolar walls and cause pneumonia. Of note, an 11-month-old girl in Spain died 10 days after admission for AC infection. The patient was treated with antiparasitic and neuroinflammation-relieving medicines, including intrathecal β-lactam antibiotics, diphenylhydantoin, corticosteroids, tetramisole, and piperazine, but died, and AC worms were found in the lungs on autopsy [34]. Together with our finding that AC not only caused severe eosinophilic meningitis during the late infection phase in nonpermissive hosts but also induced fatal pneumonia during the acute and early infection stages, this report indicated that urgent attention should be given to this issue in the future. More investigations need to be carried out to elucidate AC-induced pneumonia and to support intervention in a timely manner.

Parasitic infection triggers host immune resistance, mainly via Th1- and Th2-type responses [35]. Generally, the Th1 immune response is activated upon intracellular parasite stimulation, while the Th2 immune response is activated after extracellular parasite infection [18]. In this study, we clarified that in mice, Th1 and Th2 responses were both enhanced in the acute phase of AC infection (1 dpi), characterized by upregulation of TNF-α, IL-1β, and INOS in Th1 cells and IL-6 and IL-13 in Th2 cells. However, after the acute phase, the elevated Th1 cytokines dropped to normal levels, but the elevated Th2 cytokines were persistently upregulated. In contrast to this result, the Th1 response was dominant during acute viral infection but shifted to a Th2 response during the progression from the acute to late infection stage [36,37]. This phenomenon likely occurred because the upregulation of cytokines related to Th1 cells, such as TNF-α, and INOS favored rapid pathogen clearance. Unexpectedly, in rats, we did not observe significant activation of Th1 cytokines in the acute infection phase of AC (1 dpi), while the level of the Th2 cytokine IL-6 was obviously increased. Similar to mice, in rats, after the acute infection phase, the immune response exhibited a notable Th2 bias compared to the Th1 response. Moreover, IL-6 and IL-13 are critical Th2-related cytokines, and our results indicated that in mice, the transcript levels of IL-6 and IL-13 were significantly upregulated at 1 dpi, but the protein level of IL-13 showed no change. We speculated that differences in the posttranscriptional modifications and protein stability of IL-13 at 1 dpi might lead to the differences between transcript and protein levels.

It has been reported that chronic helminth infection induces a host Th2-type response bias and that STAT6 signaling is central for this phenotype [38]. Similarly, we also reported that during the late AC infection phase (7 to 21 dpi for mice and 7 to 42 dpi for rats), the immune response in the host lung was biased toward the Th2 type. Importantly, we found that Stat3 signaling was the activated in both mouse and rat lungs throughout the infection phase. Macrophages are considered to be immune cells that can bias toward a Th2 rather than Th1 response [38]. Consistently, in our study, we also observed that in the mouse lung, macrophages were persistently enriched during the late infection phase, while T cells and DCs aggregated only transiently. In addition, sustained inflammation could result in fibrosis [29]. However, we found fibrosis only in the rat lung after 28 dpi but not in the mouse lung. The main reason, on the one hand, was that mice infected by AC died before 28 dpi and had insufficient time for fibrosis development. On the other hand, in rats at 35 dpi and 42 dpi, all adult AC worms left the brain for the lung, which strengthened the inflammatory response in the lung and ultimately led to pulmonary fibrosis.

## Conclusion

In summary, in this study, we provided the first laboratory evidence to confirm the migration route of AC L3 in nonpermissive host mice and discovered that AC larvae could induce fatal pneumonia in mice during the acute and early infection phase while interstitial pneumonia during the late infection phase; this condition was characterized by activation of Stat3/IL-6 signaling. Moreover, administration of inhibitors targeting Stat3/IL-6 could attenuate AC-induced pneumonia in nonpermissive host mice, corroborating that Stat3/IL-6 signaling mediates the AC-induced pneumonia and providing an effective candidate target for intervention of parasitic pneumonia.

## Supporting information

S1 Video The AC L3 successfully labelled with green fluorescence exhibit excellent viability. (MP4)

S2 Video The AC L3 recovered from mouse liver at 4 hours post infection of AC. (MP4)

S3 Video The AC L3 recovered from mouse lung at 8 hours post infection of AC. (MP4)

S1 Fig **(A)** The gross morphology of mouse livers at 2, 4, 8, 12 hours post infection of AC (n = 3). **(B)** The pathological configuration of mouse livers at 2, 4, 8, 12 hours post infection of AC was displayed with H&E staining (n = 3). Magnifications: 40×, 100× and 200×.
(TIF)

S2 Fig **(A-B)** The pathological configuration of mouse (A) and rat (B) brain at 1, 2, 3, 4, 5, 6, 7 days post infection of AC was displayed with H&E staining (n = 3). Magnifications: 40×, 100× and 200×.
(TIF)

S3 Fig Protein-protein interaction network analyzed by String database (https://string-db.org/) reveals that STAT3 and NF-κB plays a central role in the regulation of Th1 and Th2 inflammation response. (TIF)

S4 Fig **(A)** The representative image of mouse brain at 7, 14, 21 days post infection as shown by H&E staining (n = 3). **(B)** The relative mRNA level of IL-4, IL-6, IL-10 and IL-13 in mouse brain at 0, 7, 14, 21 dpi of AC (n = 4). **(C)** The representative image of rat brain at 7, 14, 21, 28, 35, 42 days post infection as shown by H&E staining (n = 3). **(D)** The relative mRNA level of IL-4, IL-6, IL-10 and IL-13 of rat brain at 0, 7, 14, 21 dpi of AC (n = 4).
(TIF)

S5 Fig **(A-B)** The relative mRNA levels of Th1 cytokines (TNF-α, IL-1β, INOS, IFN-γ) in mouse (A, at 0, 7, 14, 21 dpi of AC) and rat (B, at 0, 7, 14, 21, 28, 35, 42 dpi of AC) lungs were determined by RT–qPCR (n = 4). *p < 0.05, **p < 0.01, ****p < 0.0001 compared to 0 dpi.
(TIF)

S6 Fig **(A-B)** Densitometric analysis of western blot in mouse(A) and rat(B) lungs. *p < 0.05, **p < 0.01, ***p < 0.001, ****p < 0.0001 compared to 0 dpi.
(TIF)

S7 Fig **(A-B)** The relative mRNA level of CD11b, IBA1, CD3 and CD103 in mouse (A, at 0, 7, 14, 21 dpi of AC) and rat (B, at 0, 7, 14, 21, 28, 35, 42 dpi of AC) lungs (n = 4). (**C**) The relative immunofluorescence density of CD11b, IBA1, CD3 and CD103 in rat lungs at 0, 7, 14, 21, 28, 35 and 42 dpi. *p < 0.05, **p < 0.01, ***p < 0.001, ****p < 0.0001 compared to 0 dpi.
(TIF)

S8 Fig **(A-D)** Relative mRNA level of RIP3, RIP1, Caspase-3 and Caspase-8 in mouse (A) and rat (C) lungs at the indicated dpi of AC (n = 4). Cell death of mouse (B) and rat (D) pulmonary cells at different time point of AC infection was tested by flow cytometry analysis (n = 4).
(TIF)

S9 Fig **(A)** Masson staining of mouse lungs at different dpi of AC (n = 3). **(B)** Quantitative analysis of masson staining in A. **(C)** The relative mRNA level of Col1a1, Col3a1 and α-SMA in mouse lungs after AC infection (n = 4). **(D)** Protein level of TGF-β, Col1a1, Col3a1 and α-SMA in mouse lungs were exhibited by immunoblotting (n = 3). **(E)** Relative density of the indicated proteins in D. **p < 0.01, ***p < 0.001 compared to 0 dpi.
(TIF)

S1 Table RT-qPCR Primers for the indicated genes of mouse. (DOCX)

S2 Table RT-qPCR Primers for the indicated genes of rat. (DOCX)

S3 Table The antibodies used in this study. (DOCX)

S4 Table Number of recovered AC larvae from mouse liver, lung and brain at the indicated time point after AC infection. (DOCX)
