# Supplementary figures and images for: Stat3/IL-6 signaling mediates sustained pneumonia induced by Agiostrongylus cantonensis
Source: PLoS Negl Trop Dis. 2022 May 26;16(5):e0010461. doi: 10.1371/journal.pntd.0010461 (PMC9176765; doi:10.1371/journal.pntd.0010461)

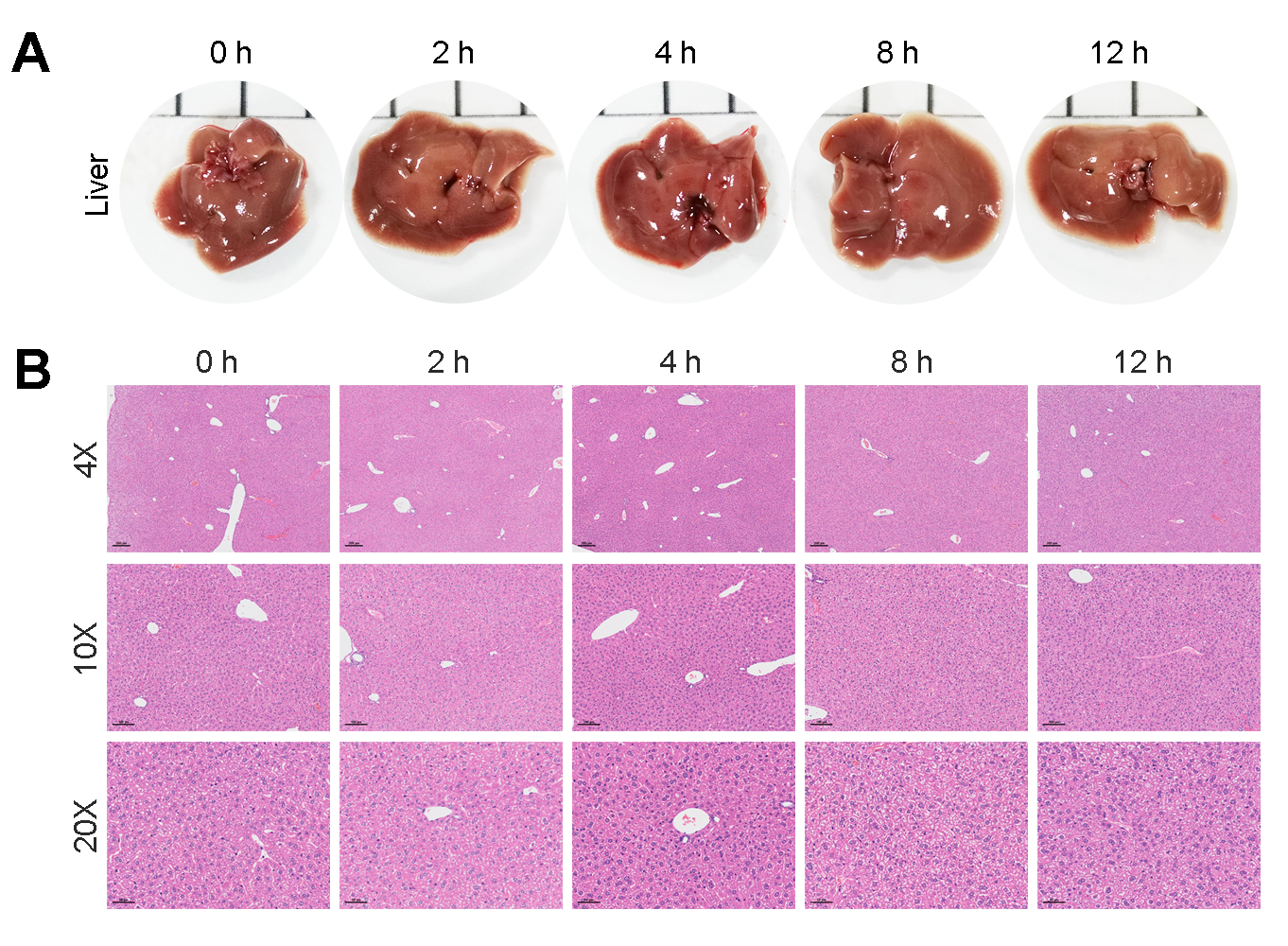

Supplement: S1 Fig — (A) The gross morphology of mouse livers at 2, 4, 8, 12 hours post infection of AC (n = 3). (B) The pathological configuration of mouse livers at 2, 4, 8, 12 hours post infection of AC was displayed with H&E staining (n = 3). Magnifications: 40×, 100× and 200×. (TIF) [file pntd.0010461.s004.tif]

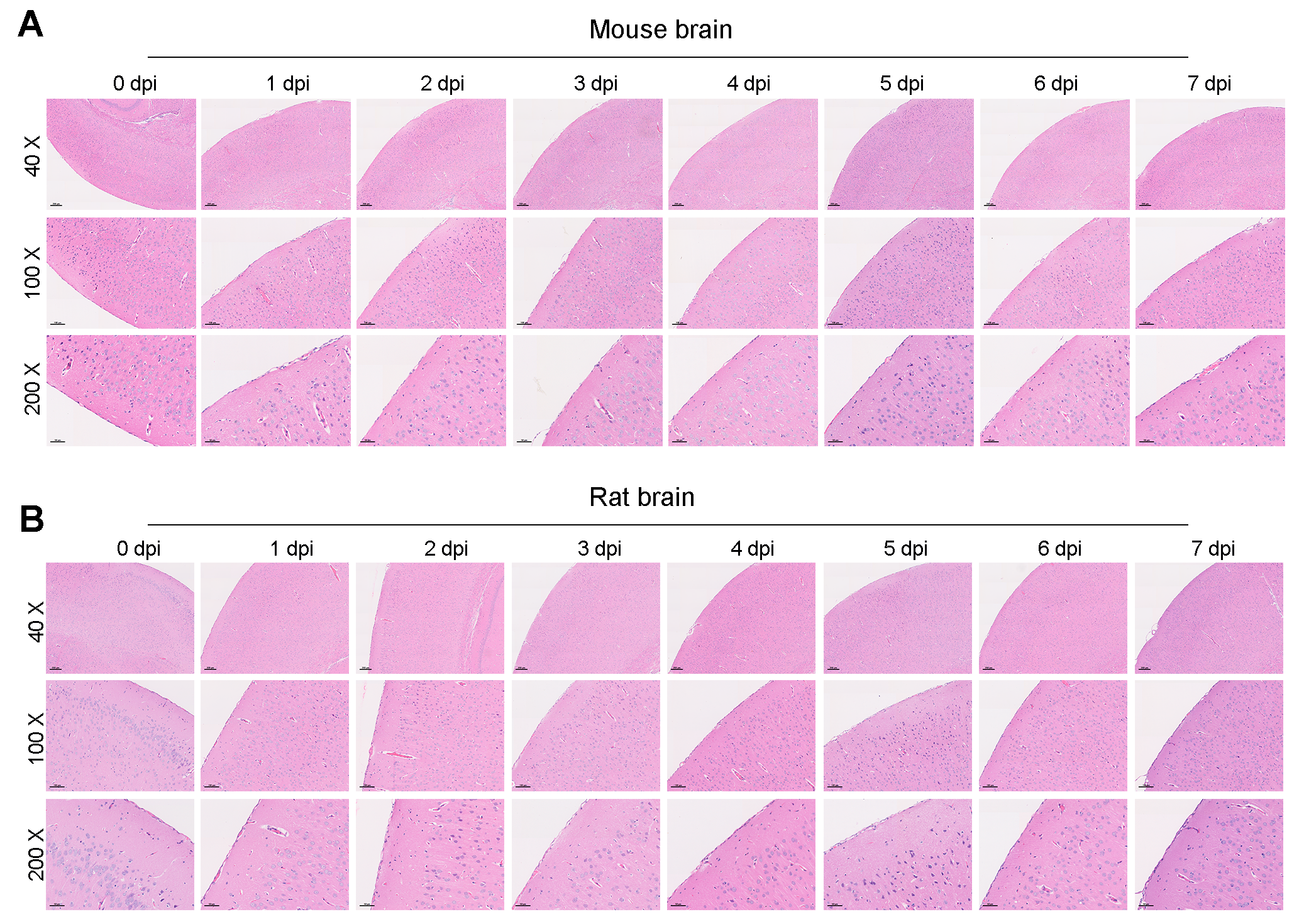

Supplement: S2 Fig — (A-B) The pathological configuration of mouse (A) and rat (B) brain at 1, 2, 3, 4, 5, 6, 7 days post infection of AC was displayed with H&E staining (n = 3). Magnifications: 40×, 100× and 200×. (TIF) [file pntd.0010461.s005.tif]

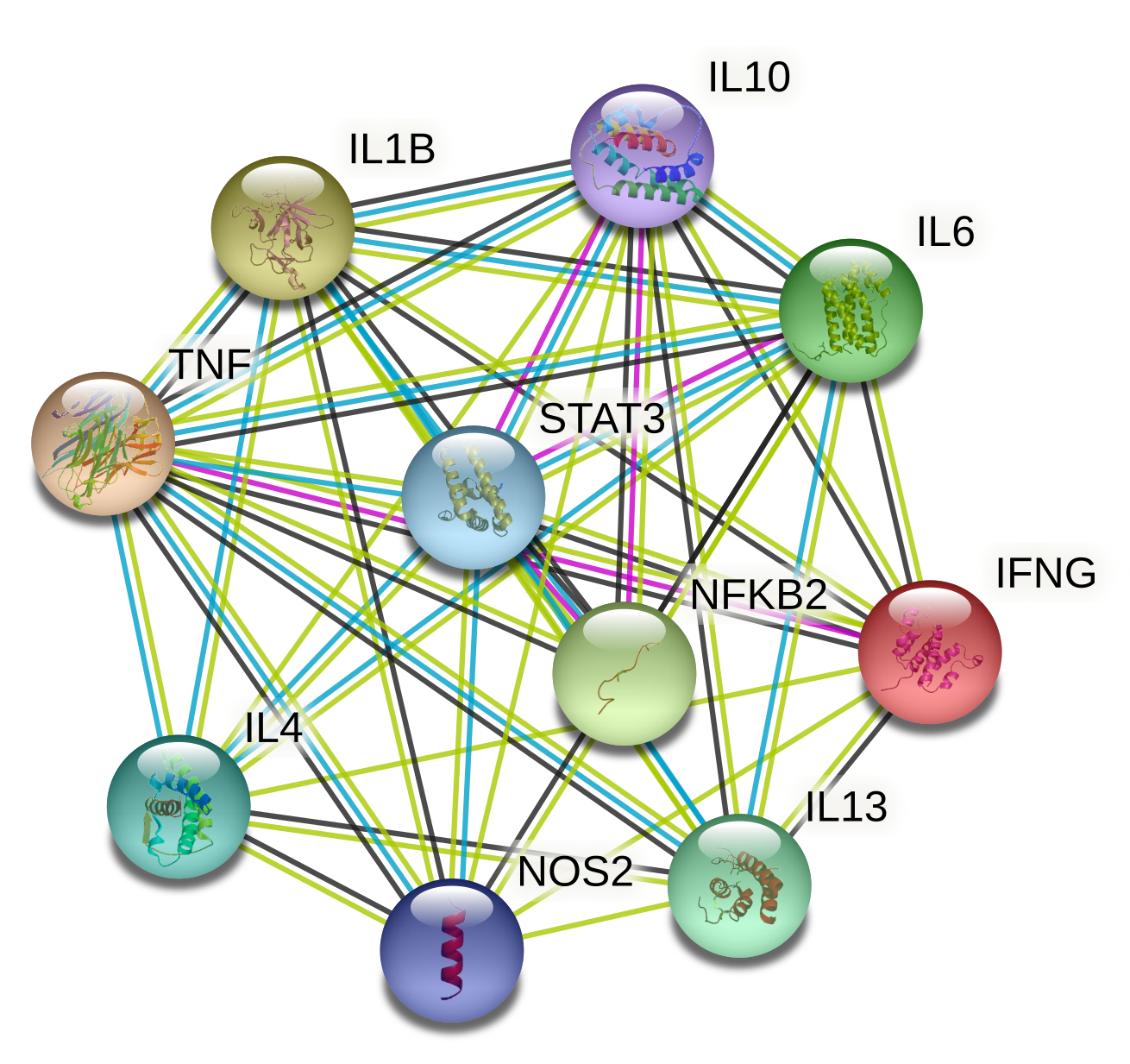

Supplement: S3 Fig — (TIF) [file pntd.0010461.s006.tif]

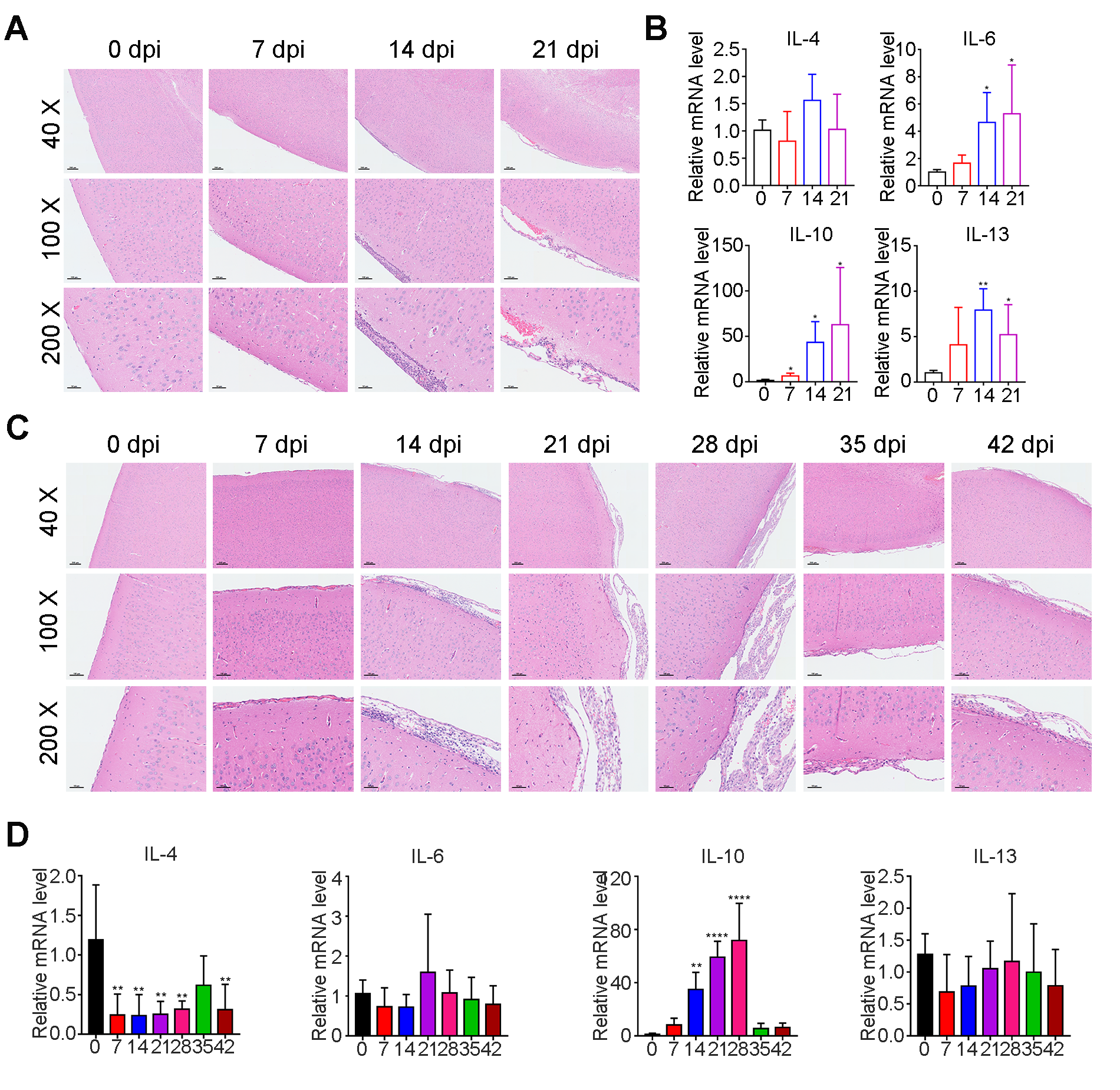

Supplement: S4 Fig — (A) The representative image of mouse brain at 7, 14, 21 days post infection as shown by H&E staining (n = 3). (B) The relative mRNA level of IL-4, IL-6, IL-10 and IL-13 in mouse brain at 0, 7, 14, 21 dpi of AC (n = 4). (C) The representative image of rat brain at 7, 14, 21, 28, 35, 42 days post infection as shown by H&E staining (n = 3). (D) The relative mRNA level of IL-4, IL-6, IL-10 and IL-13 of rat brain at 0, 7, 14, 21 dpi of AC (n = 4). (TIF) [file pntd.0010461.s007.tif]

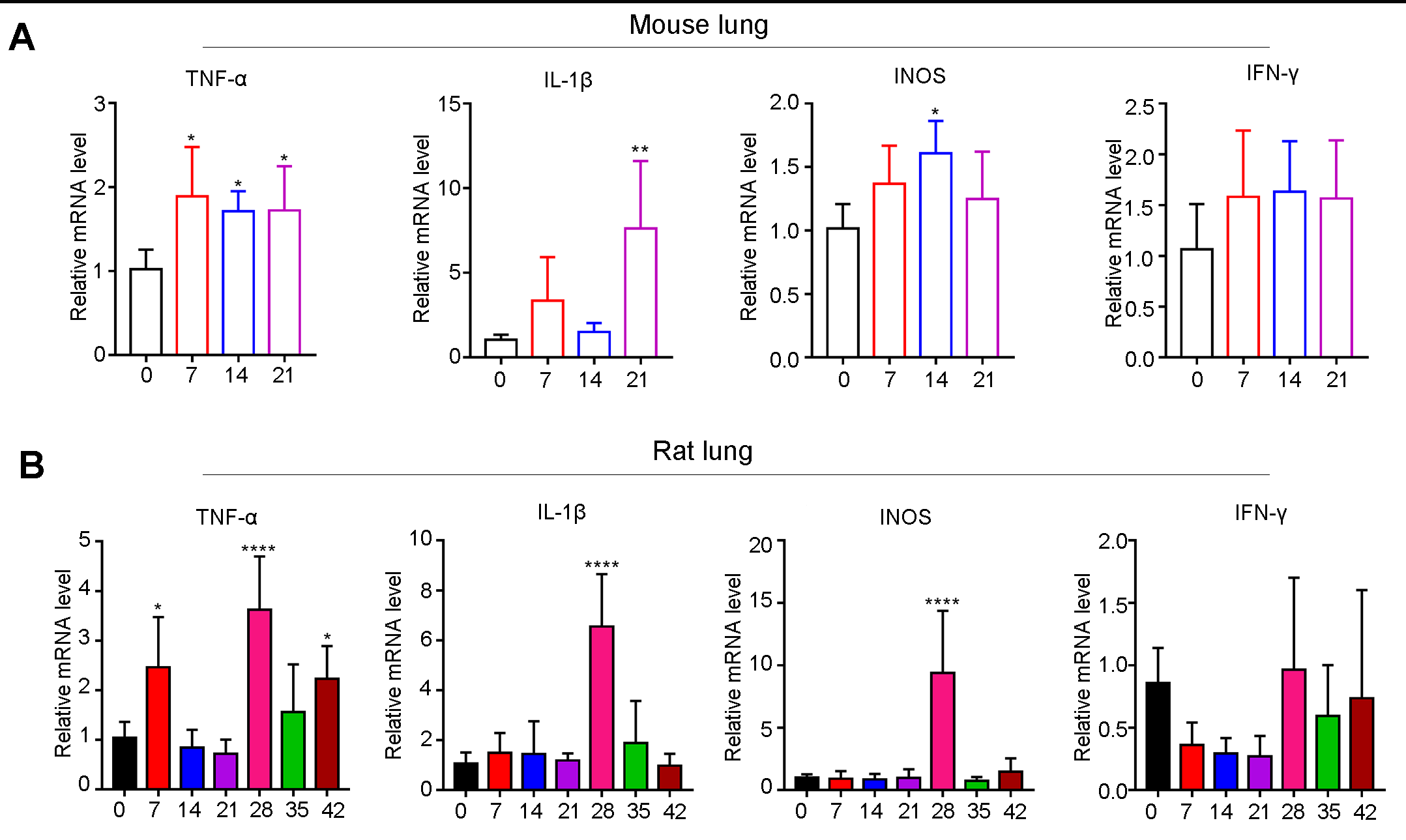

Supplement: S5 Fig — (A-B) The relative mRNA levels of Th1 cytokines (TNF-α, IL-1β, INOS, IFN-γ) in mouse (A, at 0, 7, 14, 21 dpi of AC) and rat (B, at 0, 7, 14, 21, 28, 35, 42 dpi of AC) lungs were determined by RT–qPCR (n = 4). *p < 0.05, **p < 0.01, ****p < 0.0001 compared to 0 dpi. (TIF) [file pntd.0010461.s008.tif]

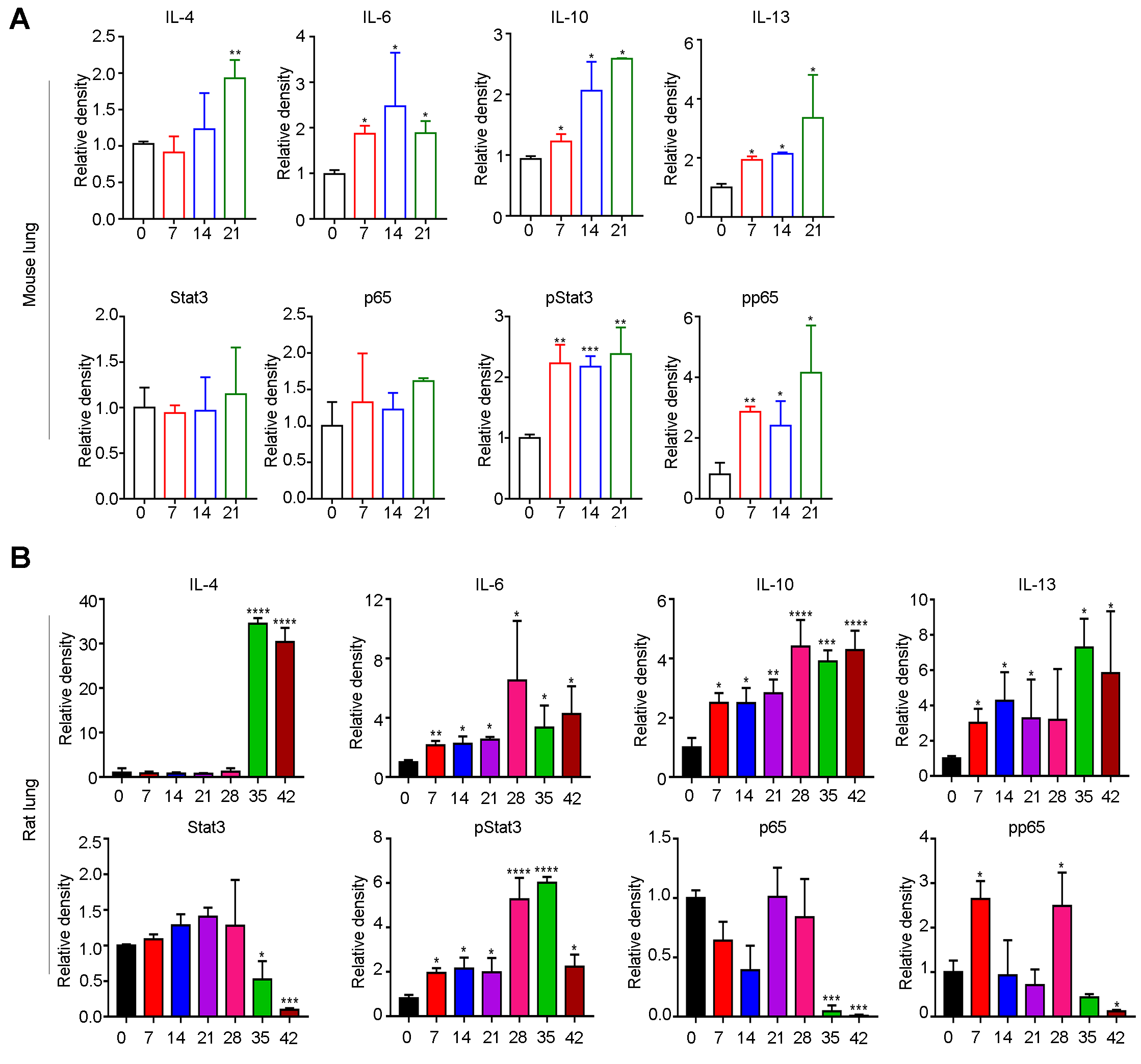

Supplement: S6 Fig — (A-B) Densitometric analysis of western blot in mouse(A) and rat(B) lungs. *p < 0.05, **p < 0.01, ***p < 0.001, ****p < 0.0001 compared to 0 dpi. (TIF) [file pntd.0010461.s009.tif]

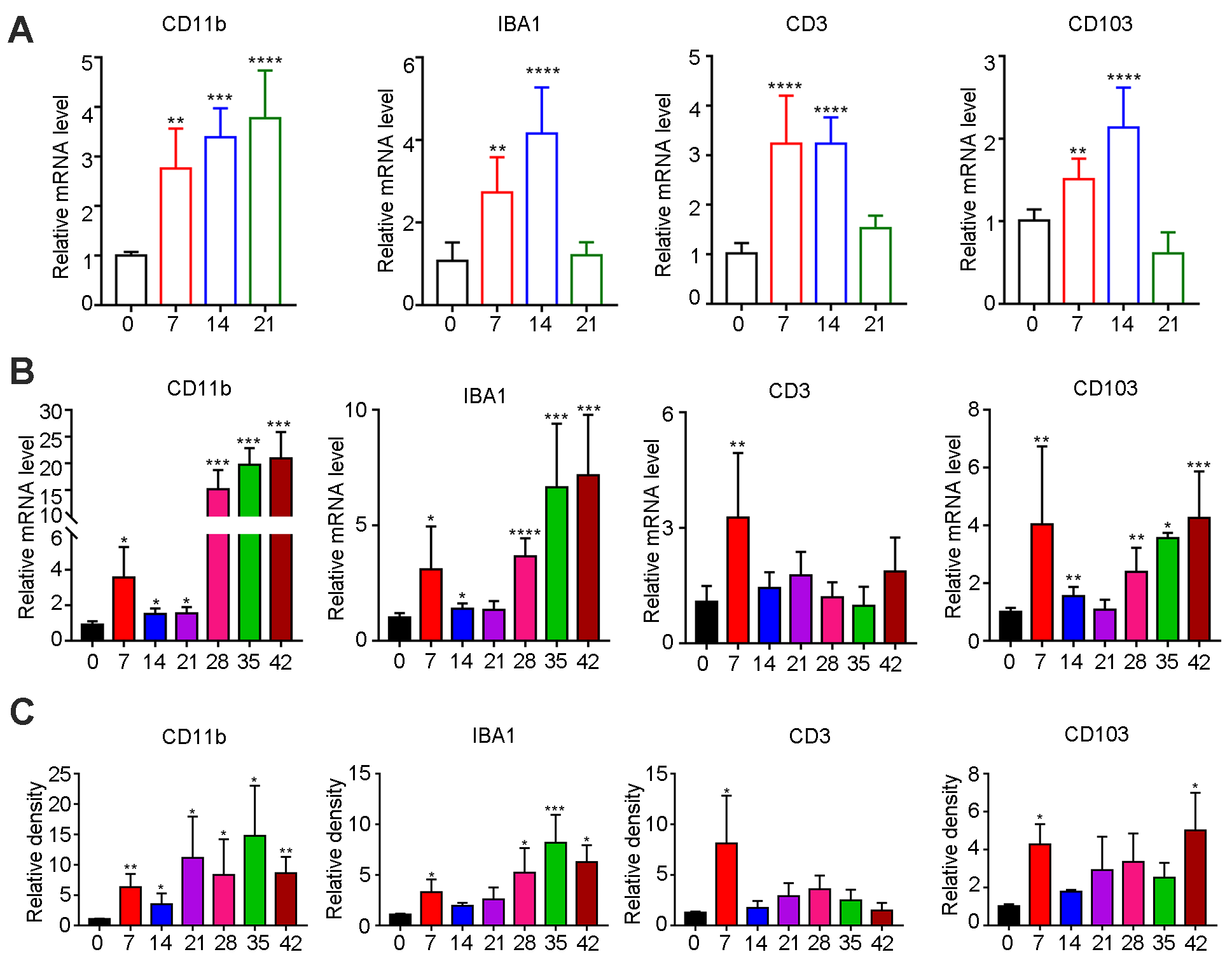

Supplement: S7 Fig — (A-B) The relative mRNA level of CD11b, IBA1, CD3 and CD103 in mouse (A, at 0, 7, 14, 21 dpi of AC) and rat (B, at 0, 7, 14, 21, 28, 35, 42 dpi of AC) lungs (n = 4). (C) The relative immunofluorescence density of CD11b, IBA1, CD3 and CD103 in rat lungs at 0, 7, 14, 21, 28, 35 and 42 dpi. *p < 0.05, **p < 0.01, ***p < 0.001, ****p < 0.0001 compared to 0 dpi. (TIF) [file pntd.0010461.s010.tif]

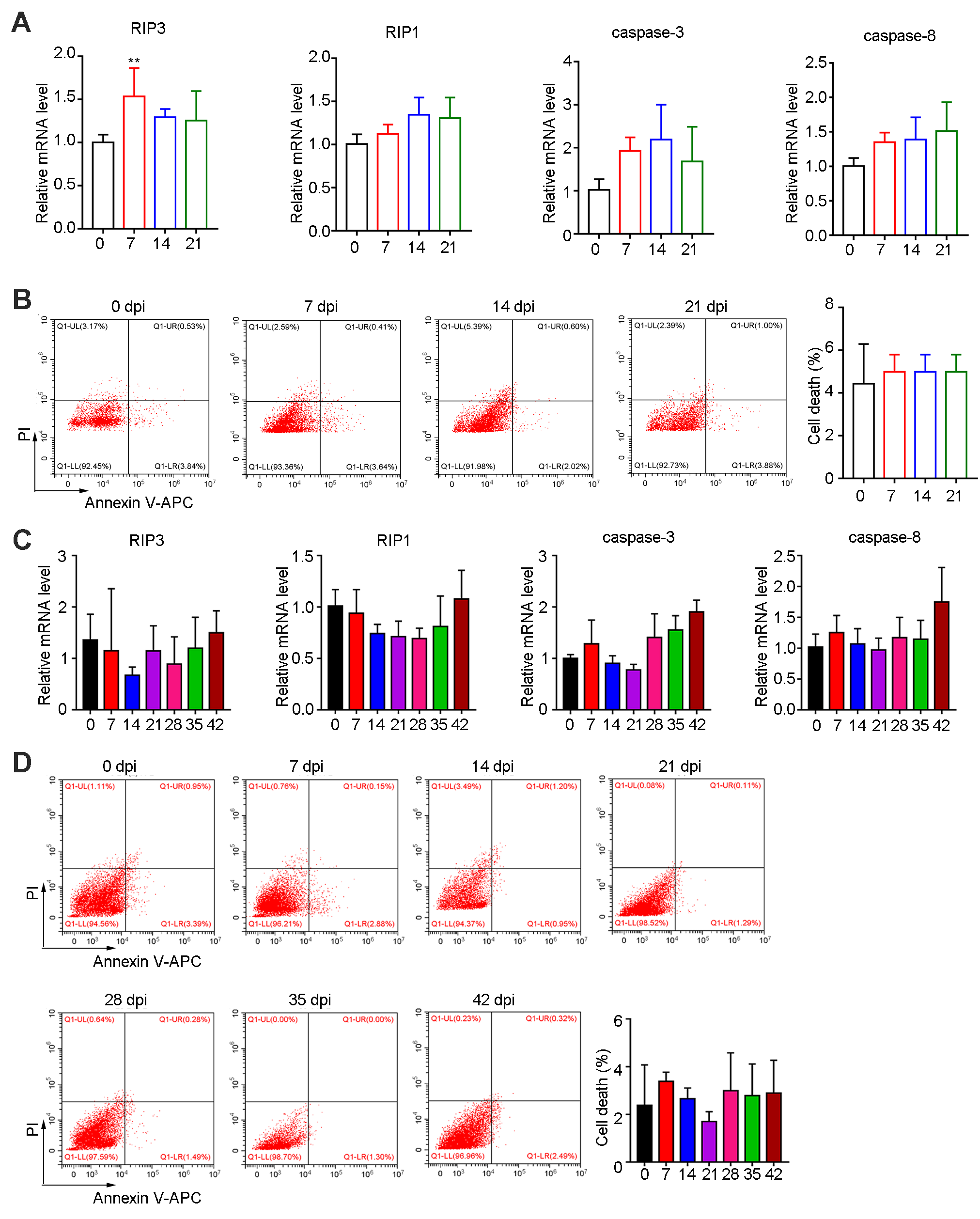

Supplement: S8 Fig — (A-D) Relative mRNA level of RIP3, RIP1, Caspase-3 and Caspase-8 in mouse (A) and rat (C) lungs at the indicated dpi of AC (n = 4). Cell death of mouse (B) and rat (D) pulmonary cells at different time point of AC infection was tested by flow cytometry analysis (n = 4). (TIF) [file pntd.0010461.s011.tif]

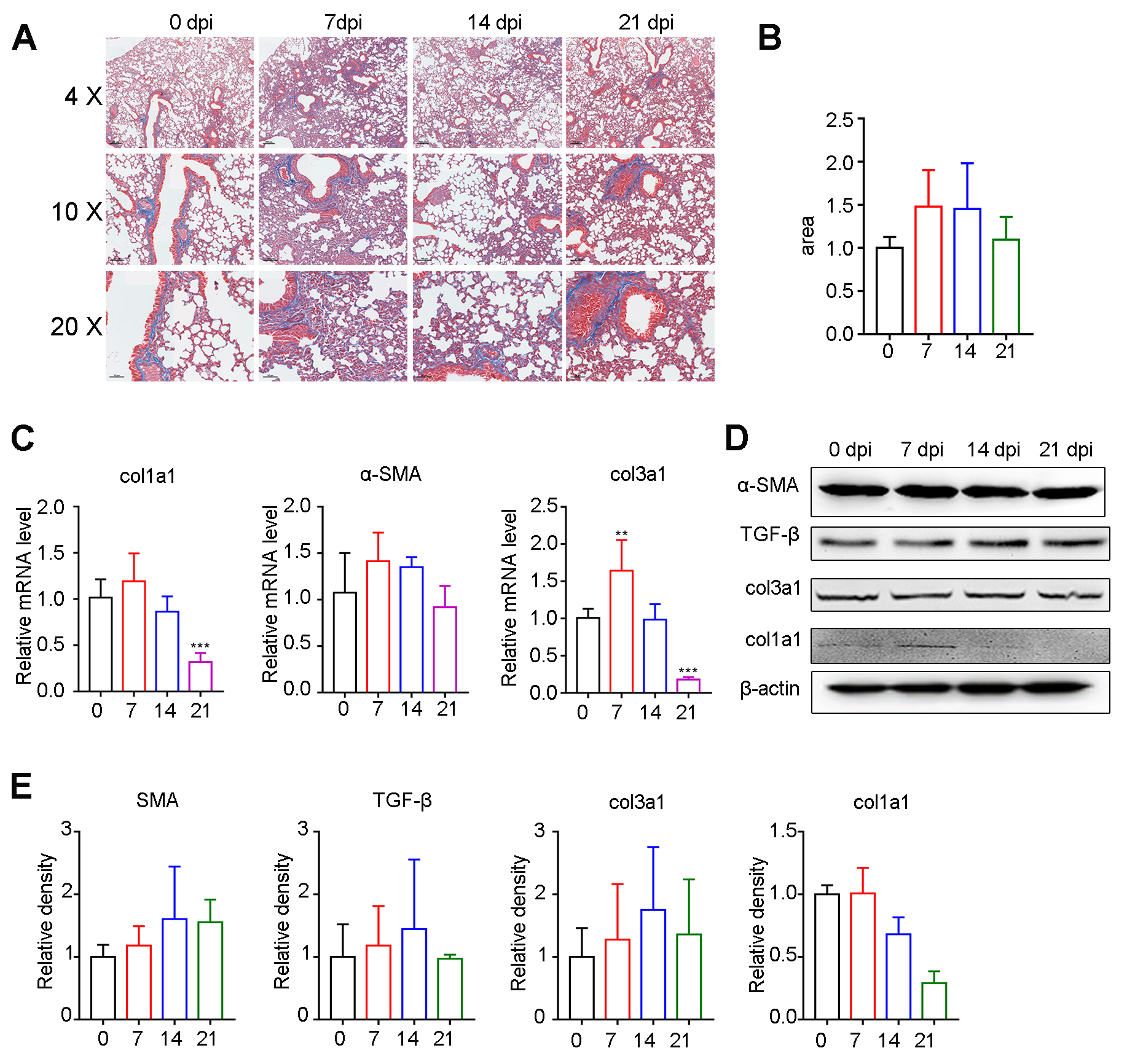

Supplement: S9 Fig — (A) Masson staining of mouse lungs at different dpi of AC (n = 3). (B) Quantitative analysis of masson staining in A. (C) The relative mRNA level of Col1a1, Col3a1 and α-SMA in mouse lungs after AC infection (n = 4). (D) Protein level of TGF-β, Col1a1, Col3a1 and α-SMA in mouse lungs were exhibited by immunoblotting (n = 3). (E) Relative density of the indicated proteins in D. **p < 0.01, ***p < 0.001 compared to 0 dpi. (TIF) [file pntd.0010461.s012.tif]
