# Supplementary material for: Stat3/IL-6 signaling mediates sustained pneumonia induced by Agiostrongylus cantonensis
Source: PLoS Negl Trop Dis. 2022 May 26;16(5):e0010461. doi: 10.1371/journal.pntd.0010461 (PMC9176765; doi:10.1371/journal.pntd.0010461)
Supplement: S1 Table — (DOCX) [file pntd.0010461.s013.docx]

**S1 Table. RT-qPCR Primers for the indicated genes of mouse.**

| **Gene symbol** | **Forward primer sequence** | **Reverse primer sequence** |
| --- | --- | --- |
| β-actin | CAATAGTGATGACCTGGCCGT | AGAGGGAAATCGTGCGTGAC |
| TNF-α | CCCTCACACTCAGATCATCTTCT | GCTACGACGTGGGCTACAG |
| INOS | GTTCTCAGCCCAACAATACAAGA | GTGGACGGGTCGATGTCAC |
| IFN-γ | ACAGCAAGGCGAAAAAGGATG | TGGTGGACCACTCGGATGA |
| IL-1β | GCAACTGTTCCTGAACTCAACT | ATCTTTTGGGGTCCGTCAACT |
| IL-4 | GGTCTCAACCCCCAGCTAGT | GCCGATGATCTCTCTCAAGTGAT |
| IL-6 | TAGTCCTTCCTACCCCAATTTCC | TTGGTCCTTAGCCACTCCTTC |
| IL-10 | GCTCTTACTGACTGGCATGAG | CGCAGCTCTAGGAGCATGTG |
| IL-13 | CCTGGCTCTTGCTTGCCTT | GGTCTTGTGTGATGTTGCTCA |
| α-SMA | CACAGCCCTGGTGTGCGACAAT | TTGCTCTGGGCTTCATCCCCCA |
| col1a1 | TCCTGCGCCTAATGTCCACCGA | AAGCGACTGTTGCCTTCGCCTC |
| col3a1 | TCCTGGTGGCAAGGGTGATCGT | TGGAGCACCAGAAGGACCAGCA |
| CD3 | ATGCGGTGGAACACTTTCTGG | GCACGTCAACTCTACACTGGT |
| CD103 | CCTGTGCAGCATGTAAAAGAATG | CAAGGATCGGCAGTTCAGATAC |
| IBA1 | ATCAACAAGCAATTCCTCGATGA | CAGCATTCGCTTCAAGGACATA |
| CD11b | GGGAGGACAAAAACTGCCTCA | ACAACTAGGATCTTCGCAGCAT |
| RIP1 | GAAGACAGACCTAGACAGCGG | CCAGTAGCTTCACCACTCGAC |
| RIP3 | AAGTGCAGATTGGGAACTACAACTC | AGAATGTTGTGAGCTTCAGGAAGTG |
| Caspase-3 | TGGTGATGAAGGGGTCATTTATG | TTCGGCTTTCCAGTCAGACTC |
| Caspase-8 | TGCTTGGACTACATCCCACAC | TGCAGTCTAGGAAGTTGACCA |
