# Supplementary material for: Stat3/IL-6 signaling mediates sustained pneumonia induced by Agiostrongylus cantonensis
Source: PLoS Negl Trop Dis. 2022 May 26;16(5):e0010461. doi: 10.1371/journal.pntd.0010461 (PMC9176765; doi:10.1371/journal.pntd.0010461)
Supplement: S2 Table — (DOCX) [file pntd.0010461.s014.docx]

**S2 Table. RT-qPCR Primers for the indicated genes of rat.**

| **Gene symbol** | **Forward primer sequence** | **Reverse primer sequence** |
| --- | --- | --- |
| β-actin | GCTACAGCTTCACCACCACA | GCCATCTCTTGCTCGAAGTC |
| TNF-α | CAATGGCATGGATCTCAAAG | TCGTCCCTTGAAGAGAACCT |
| INOS | GAGACAGGAAAGTCGGAAGC | GTGTTGAAGGCGTAGCTGAA |
| IFN-γ | AGGCCATCAGCAACAACATA | AGCTTTGTGCTGGATCTGTG |
| IL-1β | CGACAAAATCCCTGTGGCCT | TGTTTGGGATCCACACTCTCC |
| IL-4 | CCACGGAGAACGAGCTCATC | GAGAACCCCAGACTTGTTCTTCA |
| IL-6 | GTTGCCTTCTTGGGACTGAT | TGAAGTCTCCTCTCCGGACT |
| IL-10 | CCCAGAAATCAAGGAGCATT | TCACTCTTCACCTGCTCCAC |
| IL-13 | TTGCAATTGGAGATGTTGGT | TTGGTTACAGAGGCCATTCA |
| α-SMA | GTGCTGTCCCTCTATGCCTCTGG | GGCACGTTGTGAGTCACACCATC |
| col1a1 | GTACATCAGCCCAAACCCCAAG | CGGAACCTTCGCTTCCATACTC |
| col3a1 | TGATGGGATCCAATGAGGGAG | GAGTCTCATGGCCTTGCGTGTTT |
| CD3 | ACAGAGGAGCTGGCGAAGGAG | GCAGAGTGGCGATGAGATCAGTG |
| CD103 | GGTGCATCTGTGAACTCTGGTGAC | GCTTCCTCCTCGTCTTCCTCCTC |
| IBA1 | GCATCAGTAGCCAGCATCAGTACC | GTCCATCAGGCCGTCCATTGTG |
| CD11b | CTAAGGCCACCAGCGTCTGA | AGCTTTTCCTCCCTGCAAATCC |
| RIP1 | TCCTCGTTGACCGTGAC | GCCTCCCTCTGCTTGTT |
| RIP3 | GTGGGATGATGACGACG | TACGACCAGAGGCATACAGG |
| Caspase-3 | GGACCTGTGGACCTGAAAAA | GCATGCCATATCATCGTCAG |
| Caspase-8 | CTGGGAAGGATCGACGATTA | TGGTCACCTCATCCAAAACA |
