# Supplementary material for: Stat3/IL-6 signaling mediates sustained pneumonia induced by Agiostrongylus cantonensis
Source: PLoS Negl Trop Dis. 2022 May 26;16(5):e0010461. doi: 10.1371/journal.pntd.0010461 (PMC9176765; doi:10.1371/journal.pntd.0010461)
Supplement: S3 Table — (DOCX) [file pntd.0010461.s015.docx]

**S3 Table. The antibodies used in this study.**

| **Antibodies** | | **Product Brand** | | **Catalog number** | |
| --- | --- | --- | --- | --- | --- |
| Stat3 (124H6) Mouse mAb | Cell Signaling Technology | | #9139 | | |
| Phospho-Stat3 (Tyr705) (D3A7) XP® Rabbit mAb | Cell Signaling Technology | | #9145 | | |
| NF-κB p65 (D14E12) XP® Rabbit mAb | Cell Signaling Technology | | #8242 | | |
| Phospho-NF-κB p65 (Ser536) (93H1) Rabbit mAb | Cell Signaling Technology | | #3033 | | |
| Anti-IL-4 | Bioss | | bs-0581R | | |
| Anti-IL-6 | Bioss | | bs-0782R | | |
| Anti-IL-10 | Bioss | | bs-0698R | | |
| Anti-TGF beta 1 antibody [EPR21143] | Abcam | | ab215715 | | |
| Anti-alpha smooth muscle Actin antibody [E184] | Abcam | | ab32575 | | |
| COL1A1 (E8I9Z) Rabbit mAb | Cell Signaling Technology | | #91144 | | |
| Collagen Type III (N-Terminal) Polyclonal Antibody | Proteintech | | 22734-1-AP | | |
| Anti-CD3 epsilon antibody [CAL57] | Abcam | | ab237721 | | |
| Anti-CD103 antibody [EPR22590-27] | Abcam | | ab224202 | | |
| Anti-CD11b antibody [EPR1344] | Abcam | | ab133357 | | |
| Anti-Iba1 antibody [EPR16589] | Abcam | | ab178847 | | |
| GAPDH Antibody | Abways technology | | AB0036 | | |
| β-Actin (8H10D10) Mouse mAb | Cell Signaling Technology | | #3700 | | |
| Anti-mouse IgG, HRP-linked Antibody | Cell Signaling Technology | | #7076 | | |
| Anti-rabbit IgG, HRP-linked Antibody | Cell Signaling Technology | | #7074 | | |
| Anti-rabbit IgG (H+L), F(ab')2 Fragment  (Alexa Fluor® 594 Conjugate) | Cell Signaling Technology | | #8889 | | |
| Anti-rabbit IgG (H+L), F(ab')2 Fragment  (Alexa Fluor® 488 Conjugate) | Cell Signaling Technology | | | | #4412 |
