# Supplementary material for: Stat3/IL-6 signaling mediates sustained pneumonia induced by Agiostrongylus cantonensis
Source: PLoS Negl Trop Dis. 2022 May 26;16(5):e0010461. doi: 10.1371/journal.pntd.0010461 (PMC9176765; doi:10.1371/journal.pntd.0010461)
Supplement: S4 Table — (DOCX) [file pntd.0010461.s016.docx]

**S4 Table. Number of recovered AC larvae from mouse liver, lung and brain at the indicated time point after AC infection.**

| **Time (hours)** | **Liver (larvae)** | | | **Lung (larvae)** | | | | **Brain (larvae)** | |
| --- | --- | --- | --- | --- | --- | --- | --- | --- | --- |
| 0 | | 0 | | | | | 0 | | 0 |
| 2 | | 30 | | | | | 2 | | 0 |
| 4 | | | 62 | | | 8 | | | 0 |
| 8 | | | 5 | | | 31 | | | 5 |
| 12 | | 3 | | | 9 | | | | 7 |
